# Sphingolipid Remodeling and Extracellular Vesicle Signatures Reflect Disease Severity in Facioscapulohumeral Dystrophy Driven by Mitochondrial Dysfunction and Endoplasmic Reticulum Stress

**DOI:** 10.3390/antiox15091166

**Published:** 2026-09-14

**Authors:** Manuela Moriggi, Lucia Ruggiero, Enrica Torretta, Dario Zoppi, Beatrice Arosio, Chiara Fiorillo, Vincenzo Nigro, Daniele Capitanio, Cecilia Gelfi

**Affiliations:** 1 Laboratory of Proteomics and Lipidomics, IRCCS Galeazzi-Sant’Ambrogio Hospital, 20157 Milan, Italy; manuela.moriggi@grupposandonato.it (M.M.); enrica.torretta@unimi.it (E.T.); 2 Department of Neurosciences, Reproductive and Odontostomatological Sciences, University of Naples “Federico II”, 80131 Naples, Italy; lucia.ruggiero@unina.it (L.R.); d.zoppi@studenti.unina.it (D.Z.); 3 Department of Clinical Sciences and Community Health, University of Milan, 20122 Milan, Italy; beatrice.arosio@unimi.it; 4 Child Neuropsychiatryc Unit, IRCCS Istituto Giannina Gaslini, DINOGMI-University of Genova, 16147 Genova, Italy; chiara.fiorillo@edu.unige.it; 5 Department of Precision Medicine, University of Campania “Luigi Vanvitelli”, 80138 Naples, Italy; vincenzo.nigro@unicampania.it; 6 Department of Biomedical Sciences for Health, University of Milan, 20133 Milan, Italy; daniele.capitanio@unimi.it

**Keywords:** facioscapulohumeral muscular dystrophy (FSHD), lipotoxicity, mitophagy, extracellular vesicles, sphingolipid metabolism

## Abstract

Background: Facioscapulohumeral muscular dystrophy (FSHD) is a progressive and heterogeneous disorder lacking reliable circulating biomarkers for disease monitoring and therapeutic response. We hypothesized that metabolic dysregulation, lipotoxicity, and endoplasmic reticulum (ER) stress induce sphingolipid remodeling that is reflected in serum and correlates with disease severity. Methods: We performed RNA sequencing, differential expression, and pathway analyses on biceps brachii muscle biopsies from mild (*n* = 9) and severe (*n* = 5) FSHD patients and healthy controls (*n* = 6). Selected transcripts were validated by OpenArray quantitative PCR. Serum samples from mild (*n* = 9), severe (*n* = 13), and control subjects (*n* = 10) were analyzed by targeted and untargeted LC-MS/MS sphingolipidomics. Results: Severe FSHD exhibited transcriptomic signatures of mitochondrial dysfunction, ER stress, inflammation, and extracellular vesicle biogenesis. RNA-seq revealed activation of the de novo ceramide synthesis pathway, with increased SPTLC1–3, CERS2, CERS5, and DEGS1, reduced SMPD1/4 and SMPDL3A, and dysregulation of cerebroside metabolism. Migrasome/extracellular vesicle markers (TSPAN4, TM4SF1, PIGK, CPQ, ITGA5, and ITGB1) were predominantly upregulated in patients with severe disease. Serum lipidomics showed severity-dependent increases in dihydroceramides, ceramides, sphingomyelins, and dihydrosphingomyelins, while the Cer/HexCer d18:1/18:0 ratio progressively increased with disease severity. Conclusions: Integrated transcriptomic and lipidomic analyses identify sphingolipid dysregulation and extracellular vesicle biogenesis as hallmarks of severe FSHD and support circulating sphingolipids as candidate biomarkers for disease severity and therapeutic monitoring.

## 1. Introduction

Facioscapulohumeral dystrophy (FSHD) results from the activity of the double homeobox 4 (DUX4) gene, which becomes epigenetically de-repressed in affected individuals, leading to atrophy and muscle weakness in specific muscle groups, such as the face, shoulder girdle, and upper arms [1].

FSHD is also characterized by a profound clinical heterogeneity and a broad phenotypic spectrum, encompassing variations in age of onset, rate of progression, and the specific muscle groups affected. Not all muscles are affected, and not all muscles exhibiting severe inflammation undergo fat replacement [2,3], reflecting the intricate genetic mechanisms underlying the disease [4,5]. This inherent variability poses a significant challenge for patient stratification in clinical trials and often confounds the interpretation of therapeutic efficacy. Given that FSHD typically follows a slow, stepwise progression, traditional functional scales may lack the gradualness required to capture subtle disease shifts [6].

In this context, identifying robust biomarkers, particularly those reflecting metabolic dysregulation and oxidative stress, is of paramount importance. Such biomarkers would enable more accurate patient profiling and provide objective measures of disease severity and treatment response, representing a critical advance toward developing targeted therapies [6,7].

However, the identification of serum biomarkers through untargeted approaches remains challenging. Across numerous studies, lists of potential biomarkers linked to FSHD severity and progression vary widely. Analyses of gene expression in peripheral blood cells from FSHD patients compared with controls have failed to detect DUX4 target genes or to identify paired box 7 (PAX7)-regulated genes [8]. By contrast, Banerji et al. refined their analysis to reveal correlations between specific PAX7 target genes in peripheral blood mononuclear cells (PBMCs) from older FSHD patients [9].

Furthermore, recent work reports increased expression of certain miRNAs [10,11]. Targeted investigations have identified inflammatory proteins such as interleukin-6 (IL-6) and tumor necrosis factor (TNF) [12,13], as well as complement factors [14], that differ between FSHD patients and controls. However, the limited disease specificity of these markers constrains their clinical utility. A large body of previous and recent studies has focused on circulating sphingolipid signaling in blood samples from obese, insulin-resistant, and elderly individuals, highlighting the pivotal role of ceramide overproduction in these conditions [15,16,17,18,19,20,21,22,23].

However, the aim of this study is to investigate the relationship between the metabolic dysregulation observed in patients and sphingolipid signaling, which is likely driven by skeletal muscle dysmetabolism and lipotoxicity, in FSHD patients classified as having mild or severe disease according to their clinical severity score. Quantitative whole-body muscle MRI represents one of the most robust approaches and has been shown to correlate with disease activity [2,24,25,26], providing muscle-specific measures such as volume, fat fraction, and edema [27]. Radiological changes often take a long time to become evident, requiring large sample sizes for robust analysis. It may be advantageous to implement imaging sensitivity with a combined search for circulating blood biomarkers. These efforts underscore the need for well-founded hypotheses and reliable methodologies for quantifying biomarkers in the complex blood matrix.

A recent study from our group [7] indicated that, in mild FSHD patients classified according to the FSHD clinical score [28], skeletal muscle energy production is supported by an activated pentose phosphate pathway (PPP) and a functional oxidative TCA cycle, along with preserved activities of mitochondrial complexes III and IV, ATP/ADP transporters, NADPH levels, and lysosomal activity. In contrast, patients with severe FSHD exhibit a reductive PPP and TCA cycle, significant respiratory chain inhibition (> 50%), activation of reductive stress, NAD/NADPH imbalance, and impairment of autophagy and lysosomal activity. Notably, results reveal a profound remodeling of lipid metabolism in FSHD skeletal muscle, with alterations becoming progressively more pronounced as disease severity increases. Overall, the data indicate a reduction in proteins involved in fatty acid β-oxidation, accompanied by an increase in proteins associated with lipid metabolism, storage, and transport, suggesting a metabolic shift from an oxidative phenotype toward lipid accumulation that may contribute to lipotoxicity, mitochondrial dysfunction, and progressive muscle degeneration.

A recent study [29] demonstrated that antioxidant supplementation, based on the synergistic effect of vitamin C, vitamin E, zinc, and selenium, improved muscle strength and reduced oxidative stress. In the aforementioned study, an interesting observation was provided by results from transmission electron microscopy (TEM) of the cytoskeletal ultrastructural organization of vastus lateralis. The study indicated that, in some patients, affected muscles showed granular material indicative of accumulation of lipotoxic species and abnormally high-density mitochondria, leading to intermyofibrillar cohesion breakdown and a “keel” appearance.

In the current study, we hypothesize that metabolic dysregulation, lipotoxicity, and endoplasmic reticulum (ER) stress generate signaling pathways involving sphingolipids located in muscle lipid rafts of the ER and Golgi apparatus, inducing extracellular vesicle (EV) formation containing waste products, including damaged mitochondria. To test this hypothesis, we analyze the remaining muscle extracts from our previous proteomic study by RNA sequencing, together with serum samples from current participants, to associate molecular changes observed in muscle tissue with serological dysregulation of lipid species. The final goal is to identify circulating molecules reflecting altered sphingolipid signaling generated in the ER/Golgi and associated with disease severity, with a specific focus on the interplay between fatty acid metabolism dysregulation, inflammation, ER stress, and sphingolipid levels. Notably, the role of sphingolipid-regulating enzymes in ER stress signaling remains unaddressed in FSHD.

## 2. Materials and Methods

### 2.1. Study Design and Participants

This was a cross-sectional, exploratory, single-center study, conducted in accordance with the Declaration of Helsinki and approved by the Ethical Committee of the University of Naples “Federico II” (protocols 11/2012 and 30/2026). Written informed consent was obtained from all participants, including controls, before any study procedure.

Inclusion criteria were: (i) age ≥ 18 years; (ii) molecularly confirmed FSHD1, defined as a contraction of the D4Z4 repeat array to 1–10 units on a 4qA permissive haplotype; (iii) written informed consent. Exclusion criteria were: (i) any other neuromuscular, autoimmune, inflammatory, hepatic or renal disease; (ii) diabetes mellitus and treatment with lipid-lowering, corticosteroid or immunosuppressive drugs, all of which modify the circulating sphingolipidome; (iii) acute infection or febrile illness within the four weeks preceding sampling; (iv) pregnancy. Disease severity was graded with the FSHD clinical score, a standardized sum score (range 0–15) of the degree of involvement of six independent muscle districts [28], and each patient was assigned a clinical category according to Ricci et al. Patients were allocated to the mild group when the FSHD clinical score was ≤5 and to the severe group when it was ≥6.

Anthropometric measurements, routine biochemistry, and clinical scores of all participants are reported in Supplementary Information Table S1, where each parameter is given with its unit of measurement and the reference interval of the laboratory that performed the analysis. Routine biochemistry had been obtained as part of standard clinical care and was not available for every parameter in every participant; the number of subjects available for each parameter is stated in Table S1 (triglycerides *n* = 19, HDL cholesterol *n* = 21, LDL cholesterol *n* = 17, total cholesterol *n* = 20, ESR *n* = 18, CRP *n* = 20 of 22 patients). The two patient groups were comparable for age (mild 42.8 ± 17.8 vs. severe 49.0 ± 17.6 years, *p* = 0.43) and body mass index (21.7 ± 4.3 vs. 24.8 ± 4.4 kg/m^2^, *p* = 0.13).

### 2.2. Muscle Biopsies

Muscle biopsies from the biceps brachii of 9 mild and 5 severe FSHD patients and 6 controls were from our previously published cohort [7]. This is an independent cohort, distinct from the serum cohort of the present study, whose FSHD clinical scores were overall distributed toward higher severity. Accordingly, in that study, the mild/severe boundary was set at a higher value, with mild patients ranging from 4 to 7 and severe patients from 8 to 15. In the present serum study, conducted on a separate cohort, a more restrictive stratification was adopted (mild ≤ 5, severe ≥ 6). Individual FSHD clinical scores of the biopsy cohort are reported in Supplementary Table S1 [7].

### 2.3. Serum Samples

Peripheral blood samples were collected from 9 FSHD mild (FSHD score 1–5), 13 FSHD severe (FSHD score 6–13), and 10 CTR subjects into Serum Separating Tubes (SST). Immediately after collection, samples were transported on ice to the respective local laboratories and processed within a short timeframe. Upon arrival, each sample was centrifuged at +4 °C for 15 min at 2000× *g*. The resulting serum was carefully aliquoted into pre-chilled sterile polypropylene microtubes and immediately stored at −80 °C until further analysis. Hemolyzed samples were excluded. No more than 45 min elapsed between blood collection and final freezing. To ensure metabolite stability, samples were not subjected to any freeze-thaw cycles prior to analysis.

Samples collected at the University of Naples “Federico II” (Naples, Italy) were subsequently shipped on dry ice within 24 h to the University of Milan (Milan, Italy). All samples were anonymized prior to long-term storage to ensure confidentiality and compliance with ethical standards.

### 2.4. RNA Sequencing in Muscle Biopsies

Total RNA was extracted from biceps brachii biopsies, and 100 ng of total RNA from each sample was used for library preparation with the TruSeq RNA Access Library Prep Kit (Illumina, San Diego, CA, USA), following the manufacturer’s instructions. Briefly, RNA was fragmented by heat treatment and reverse-transcribed into first-strand cDNA using random primers, followed by second-strand synthesis with simultaneous incorporation of sequencing adapter sequences. Coding regions of the transcriptome were then selectively enriched by hybridization with sequence-specific capture probes, generating the final sequencing libraries. Library quality and fragment size distribution were assessed using the Agilent 2100 Bioanalyzer (Agilent Technologies, Santa Clara, CA, USA), showing a predominant fragment size ranging from 230 to 270 bp.

Sequencing was performed on an Illumina HiSeq 1000 (Illumina, San Diego, CA, USA) platform using a paired-end strategy, with four libraries multiplexed per lane. A sequencing depth ranging from approximately 30 to 80 million paired-end reads per sample was achieved.

Differential gene expression analysis was performed using the edgeR package within the Bioconductor framework, applying the generalized linear model (GLM) approach [30,31]. Raw read counts were normalized according to the edgeR pipeline, and statistical significance was assessed using the Benjamini–Hochberg procedure to control the false discovery rate (FDR). Transcripts with an FDR ≤ 0.05 and an absolute fold change ≥ 2 were considered differentially expressed.

#### Ingenuity Pathway Analysis

Functional and network analyses of statistically significant transcript expression changes were performed using IPA software (Qiagen, Hilden, Germany, Summer Release 2024). The “Core Analysis” function was employed to understand the significance of the data by examining canonical pathways enriched with differentially regulated proteins. *p*-values were calculated using a right-tailed Fisher’s exact test, and the activation z-score was used to predict pathway activation or inhibition. Pathways with a Fisher’s exact test *p*-value < 0.05 and a z-score ≤ −2 or ≥ 2 were considered statistically significant.

### 2.5. Gene Expression Analysis in Muscle Biopsies

To validate selected RNA-seq findings and to further investigate the expression of selected genes involved in sphingolipid metabolism, reverse transcription quantitative PCR (RT-qPCR) was performed to confirm mRNA expression levels. Total RNA was extracted from 6–18 mg of muscle biopsies using TRItidy G™ (PanReac AppliChem, ITW reagents, Monza, Italy) according to the manufacturer’s instructions. RNA concentration was determined using a NanoPhotometer N60 spectrophotometer (Implen GmbH, München, Germany). One to two micrograms of total RNA were reverse-transcribed using the SuperScript VILOTM cDNA Synthesis Kit (Invitrogen by Thermo Fisher Scientific, Waltham, MA, USA). Quantitative PCR analysis was performed using the OpenArray^®^ QuantStudio 12K Flex Real-Time PCR System (Applied Biosystems by Thermo Fisher Scientific, Waltham, MA, USA), with commercial probes listed in Supporting Information Table S2. GAPDH, ACTB, and 18S genes were included in the OpenArray^®^ chip and used as endogenous housekeeping control genes. A total volume of 5 µL per reaction, containing 60–120 ng of cDNA (1.2 µL), 1.3 µL of PCR-grade water, and 2.5 µL of TaqMan™ OpenArray^®^ Real-Time PCR Master Mix (Applied Biosystems), was loaded for each sample. All analyses were performed in duplicate. Readouts were expressed as 2^−ΔCt^.

Statistical analysis was conducted using one-way analysis of variance (ANOVA) followed by Tukey’s post hoc tests (*p* < 0.05) for comparison between the following: CTR vs. FSHD mild, and CTR vs. FSHD severe. In cases where the ANOVA test was not applicable, the non-parametric Kruskal–Wallis test was applied. False discovery rate (FDR) correction was used to correct for multiple tests to reduce the overall error.

### 2.6. Sphingolipid Analysis

#### 2.6.1. Reagents and Chemicals

LC-MS analytical-grade water and methanol were from Thermo Fisher Scientific. LC-MS grade ammonium formate, formic acid, and acetic acid, as well as 3,5-Di-tert-4-butylhydroxytoluene (BHT), were from Sigma-Aldrich (Saint Louis, MO, USA). HPLC analytical grade ethanol and chloroform were from VWR international (Milan, Italy). Potassium hydroxide was from Merck Millipore (Burlington, MA, USA). Sphingosine (d17:1), sphinganine-1-phosphate (d17:1), ceramide (d18:1/12:0), sphingomyelin (d18:1/12:0) and glucosyl (β) ceramide (d18:1/12:0) were from Avanti Polar Lipids (Alabaster, AL, USA).

#### 2.6.2. Sphingolipid Extraction

Sphingolipids were extracted from sera of patients recruited for the present study. For extraction, 50 µL of serum was fortified with 200 pmol of each internal standard (sphingosine (d17:1), sphingosine-1-phosphate (d17:1), ceramide (d18:1/12:0), sphingomyelin (d18:1/12:0), and glucosyl (β) ceramide (d18:1/12:0)) and mixed with 0.1 mL of LC-MS water and 1.5 mL of methanol/chloroform 2:1. Samples were briefly sonicated and heated at 48 °C overnight. Then, 0.15 mL of potassium hydroxide (KOH) 1 M in methanol was added to each sample for saponification, which was neutralized with 0.15 mL of acetic acid 1 M after 2 h of incubation at 37 °C. Samples were dried, resuspended in methanol, and centrifuged for 3 min at 10,000× *g*. Supernatants were collected in UPLC glass vials and stored at −80 °C until analyses.

#### 2.6.3. Targeted Lipidomics

Ceramides (Cer), dihydroceramides (dhCer), sphingomyelins (SM), dihydrosphingomyelins (dhSM) and hexosylceramides (HexCer), sphingosine and sphingosine-1-phosphate (S1P) were detected by Multiple-Reaction Monitoring Mass Spectrometry (MRM-MS) using a Xevo TQ-S micro mass spectrometer (Waters, Milford, MA, USA). 2 µL of extract were injected and separated on a C8 Acquity UPLC BEH (Waters), 100 mm × 2.1 mm, id 1.7 µm (Waters), kept at 30° C, using the following linear gradient: 0.0 min: 15% B; 1 min: 30% B; 1.10 min: 70% B; 4.50 min: 70% B; 4.51 min: 99% B; 5 min: 99% B; 5.10 min: 15% B; 6.60 min: 15% B at 0.3 mL/min flow rate. Phase A was 2 mM ammonium formate in acetonitrile/water (60:40, *v*/*v*) with 0.1% formic acid, while phase B consisted of 2 mM ammonium formate in isopropanol/acetonitrile/water (90:9:1, *v*/*v*/*v*) with 0.1% formic acid. An electrospray interface operating in positive ion mode was employed to obtain MS/MS spectra by acquiring MRM transitions (Supporting Information Table S3). The capillary voltage was set at 3.5 kV. The source temperature was set to 150 °C. Desolvation gas flow was set to 1000, and desolvation temperature was set to 350 °C. Data were acquired by MassLynx™ 4.2 software and quantified by TargetLynx 4.2 software. Results were normalized to protein content, determined by bicinchoninic assay (BCA Pierce).

#### 2.6.4. Untargeted Lipidomics

Liquid chromatography-mass spectrometry (LC-ESI/MS) analysis of lactosylceramides (LacCer) and GM3 ganglioside was performed on a Q-TOF Synapt G2-Si (Waters) linked to a Waters Acquity UPLC system. 10 µL of sphingolipid extract were separated on a C8 Acquity UPLC BEH (Waters), 100 mm × 2.1 mm, id 1.7 µm, kept at 30° C, with the following linear gradient: 0.0 min: 80% B; 3 min: 90% B; 6 min: 90% B; 15 min: 99% B; 18 min: 99% B; 20 min: 80% B, at 0.3 mL/min flow rate. Phase A consisted of 2 mM ammonium formate in H_2_O, with 0.05 mM formic acid, while phase B was 1 mM ammonium formate in methanol, with 0.05 mM formic acid. The ESI ionization source was operated in positive ion mode with a sample cone voltage of 30 V and a capillary voltage of 3.0 kV. The desolvation temperature was set to 150 °C, and the ion source temperature was 120 °C. The desolvation gas flow was set to 600 L/h. Data were captured under centroid mode, and the range of the mass scan was set to 50–1500 Da. Accuracy and reproducibility were maintained by employing an independent reference spray via Lock-Spray. Sphingolipid quantification was carried out using the ion chromatogram obtained for each compound using 50 mDa windows. The linear dynamic range was determined by injection of standard mixtures. Positive identification of compounds was based on the accurate mass measurement, with an error <5 ppm, and their retention time, compared to that of a standard (±2%). Mass spectra were analyzed by MassLynx™ 4.2 Software (Waters), and lipids were annotated as lipid subclasses as follows (sphingosine backbone/number of carbon atoms of the fatty acid: number of unsaturations of the fatty acid). MS/MS spectra were acquired, and assignment of species was based on precursor ions and product ions *m*/*z* 264.268 and *m*/*z* 266.286, which correspond to the sphingosine backbone (d18:1) and dihydrosphingosine backbone (d18:0), respectively. Results were normalized to protein content, determined by bicinchoninic assay (Pierce, Thermo Scientific, Waltham, MA, USA).

#### 2.6.5. Statistical Analysis

Differences in Cer, dhCer, SM, dhSM, HexCer, LacCer and GM3 levels were assessed among groups, with a semiquantitative approach, by comparing the abundance of sphingolipids across samples. Statistical analysis was performed using GraphPad Prism Software v.8.0.2. We assessed data normality using the Shapiro–Wilk test (α = 0.05). When normality was confirmed, we applied ANOVA (*p* < 0.05) followed by Tukey’s post hoc test; otherwise, a non-parametric test (Friedman, *p* < 0.05) was performed, followed by Dunn’s multiple comparisons test. The coefficient of variation (CV) was assessed to be below 10%.

### 2.7. Immunoblotting of Selected Molecules in Serum

Protein extracts (50 µg) from CTR, FSHD mild, and severe serum samples were loaded and resolved on a 6–14% gradient polyacrylamide gel. Blots were incubated with antibodies bought by the Santa Cruz Biotechnology (Dallas, TX, USA), Invitrogen and Assay Genie (Dublin, Ireland): rabbit polyclonal anti-perilipin 4 (PLIN4), 1:1000; mouse monoclonal anti-apolipoprotein A1 (APOA1), 1:1000. After washing, membranes were incubated with anti-rabbit (Cytiva, Buccinasco, Italy, 1:10,000) or anti-mouse (Jackson ImmunoResearch, Ely, UK, 1:5000) secondary antibody conjugated with horseradish peroxidase. Signals were visualized by chemiluminescence using the ECL Prime detection kit and the Image Quant LAS 4000 (Cytiva) analysis system. Band quantification was performed using ImageQuant TL v. 8.1 (Cytiva), and statistical analyses were conducted with GraphPad Prism v. 8.0.2. Serum samples were analyzed by one-way ANOVA followed by Tukey’s post hoc test (*p* < 0.05). Band intensities were normalized to total protein content detected by Sypro Ruby staining.

## 3. Results

**Figure 1:**
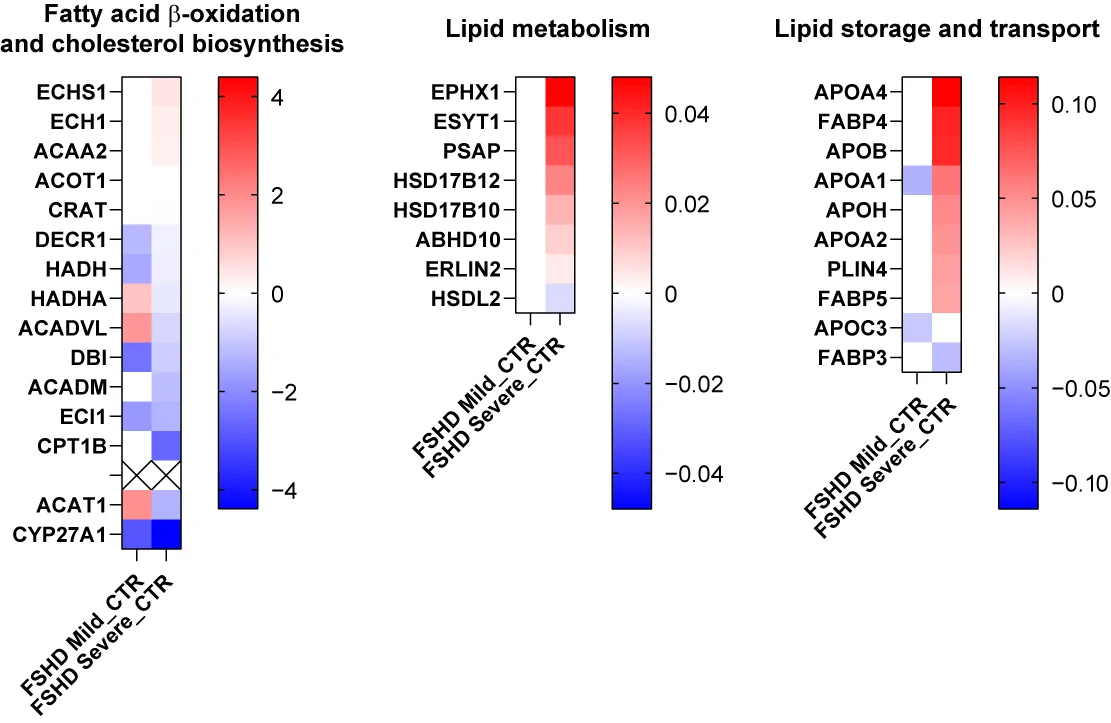
Differential abundance of lipid-related proteins in FSHD muscles. Heatmaps display changes in protein abundance across mild FSHD, severe FSHD, and matched healthy controls (CTR). Proteins are grouped by functional pathways: fatty-acid β-oxidation and cholesterol biosynthesis, lipid metabolism, and lipid storage and transport. Color gradients represent relative protein abundance log-fold changes (red = increased; blue = decreased) compared with controls.

In our previous study [7], proteomic analysis of skeletal muscle identified differential regulation of proteins involved in fatty acid β-oxidation, cholesterol biosynthesis, lipid metabolism, lipid storage, and transport in patients with mild and severe FSHD compared with controls (Figure 1). Mild FSHD was characterized by partial retention of mitochondrial fatty acid β-oxidation, whereas severe FSHD displayed a proteomic profile consistent with lipotoxicity. Patients with severe FSHD were characterized by increased levels of apolipoproteins (APOA2, APOA4, APOB, APOH) and perilipin-4 (PLIN4), alterations in proteins located in lipid rafts, together with decreased levels of acetyl-CoA acetyltransferase 1 (ACAT1) and cytochrome P450 family 27 subfamily A member 1 (CYP27A1), two enzymes involved in cholesterol homeostasis.

### 3.1. RNA-Sequencing in Muscle Biopsies

**Figure 2:**
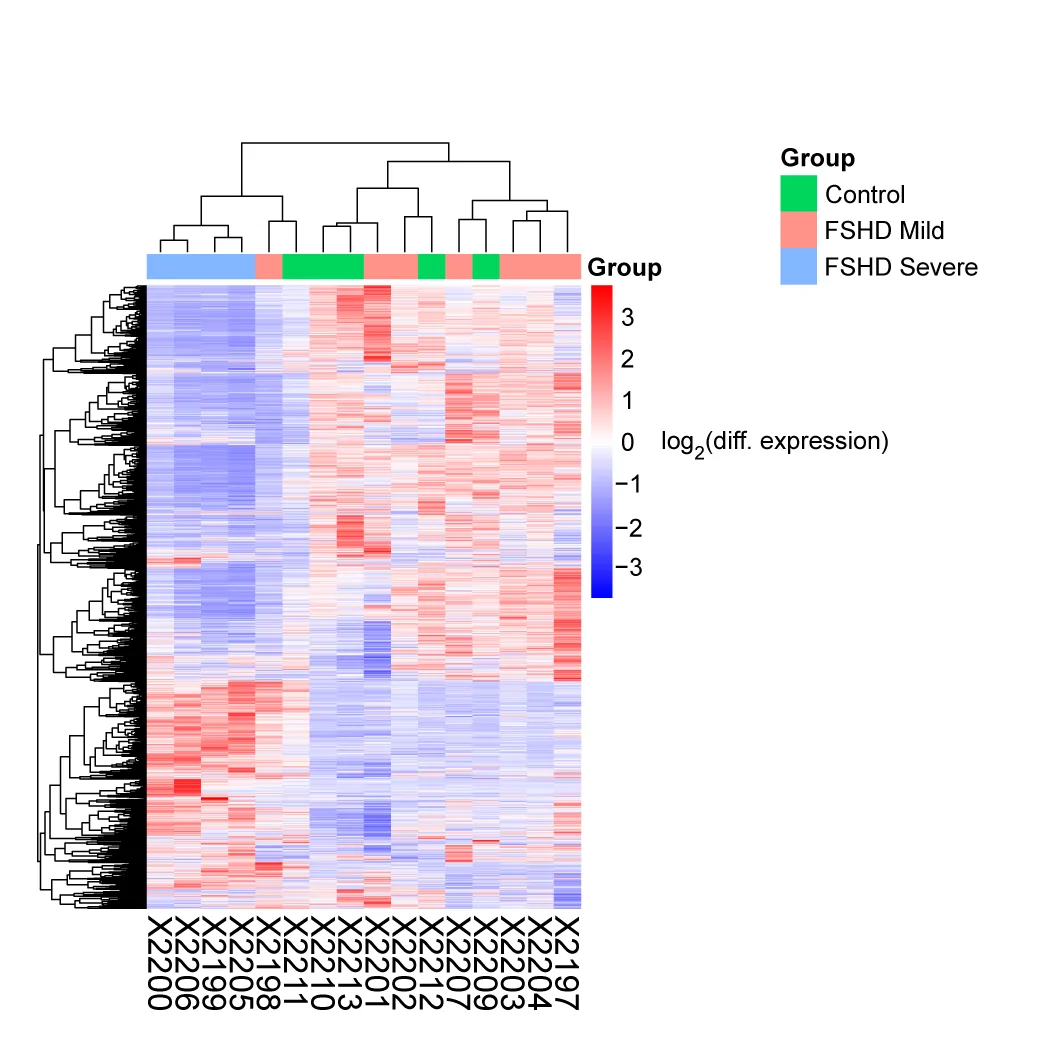
Hierarchical clustering of the 5000 RNA-seq genes with the most intense expression signals. The heatmap shows log_2_-transformed differential expression values across samples. Columns represent individual samples, and rows represent genes. Samples are annotated by group: Control (green), FSHD Mild (pink), and FSHD Severe (blue). Red indicates higher expression and blue indicates lower expression. Dendrograms illustrate the hierarchical clustering of both genes and samples based on expression patterns.

To confirm and enlarge the dataset of proteomic results, RNA-seq analysis was performed on muscle extracts from the same mild and severe patients (Supporting Information Table S4). Hierarchical clustering analysis of the 5000 most highly expressed transcripts showed that severe samples clustered together in a distinct branch of the dendrogram. This tight clustering suggests that the severe phenotype is associated with a more pronounced and homogeneous alteration of gene expression. In contrast, mild FSHD samples did not form a clearly distinct cluster, indicating that their transcriptional profile is more similar to that of healthy controls or that molecular alterations are less pronounced and more heterogeneous. Overall, clustering analysis demonstrates that clinical severity is reflected at the transcriptomic level, with severe cases showing a distinct molecular signature, whereas mild cases display an intermediate or partially overlapping expression pattern relative to controls (Figure 2).

IPA of the RNA dataset indicates 696 significantly enriched pathways (Supporting Information Table S5). Among these, the 29 with the most negative z-scores (−8 < z-score < −2) were predicted to be inhibited, whereas the 20 with the highest positive z-scores (2 < z-score < 5) were predicted to be activated (Supporting Information Figure S1). The analysis revealed a strong and coordinated inhibition of mitochondrial pathways in FSHD severe samples. In patients with severe FSHD, pathways related to mitochondrial translation, oxidative phosphorylation, respiratory electron transport chain, TCA cycle, and mitochondrial complex assembly showed negative z-scores, indicating impaired mitochondrial bioenergetics and reduced oxidative metabolism. Pathways involved in mitophagy, autophagy, and protein ubiquitination are significantly enriched, suggesting dysregulation of stress-response and protein quality control mechanisms. The presence of altered rapamycin (mTOR), kelch-like ECH-associated protein 1–nuclear factor erythroid 2-related factor 2 (KEAP1-NFE2L2), and tumor protein p53 (TP53) metabolic signaling further supports a metabolic stress condition.

In parallel, there was clear activation of inflammatory and immune-related pathways, including interleukin-1, -2, -8, -15 (IL-1, IL-2, IL-8, IL-15) signaling, the inflammasome pathway, TREM1 signaling, and neuroinflammation signaling, indicating a pronounced pro-inflammatory environment in FSHD severe muscle.

Extracellular matrix (ECM) remodeling pathways, including collagen biosynthesis, ECM organization, integrin signaling, and ECM degradation, were also significantly enriched, indicating structural tissue remodeling.

Interestingly, lipid metabolism also emerged as a significantly affected biological process. While the mitochondrial fatty acid β-oxidation pathway was predicted to be inhibited, pathways regulating fatty acyl-CoA biosynthesis and PPARα-dependent lipid metabolism were activated. The concomitant activation of lipid metabolic pathways, together with inhibition of mitochondrial fatty acid utilization, points to metabolic rewiring that may favor lipid accumulation and altered lipid remodeling in severe FSHD muscles. To date, several lipid-related pathways were significantly enriched but did not reach the z-score threshold required for reliable activation-state prediction. These included Sphingolipid Metabolism, Sphingosine-1-Phosphate Signaling, PPAR Signaling, NR1H2/NR1H3 (LXR) Signaling, Regulation of Cholesterol Biosynthesis by SREBP, Carnitine Metabolism, Lipophagy, Triglyceride Metabolism, Phosphatidylcholine Biosynthesis, CDP-Diacylglycerol Biosynthesis, and Plasma Lipoprotein Assembly, Remodeling and Clearance. Although their activation state could not be confidently inferred, their significant enrichment further supports a broad remodeling of lipid homeostasis involving fatty acid metabolism, membrane phospholipid remodeling, cholesterol regulation, lipoprotein metabolism, and sphingolipid pathways in severe FSHD (Supporting Information Table S5).

**Figure 3:**
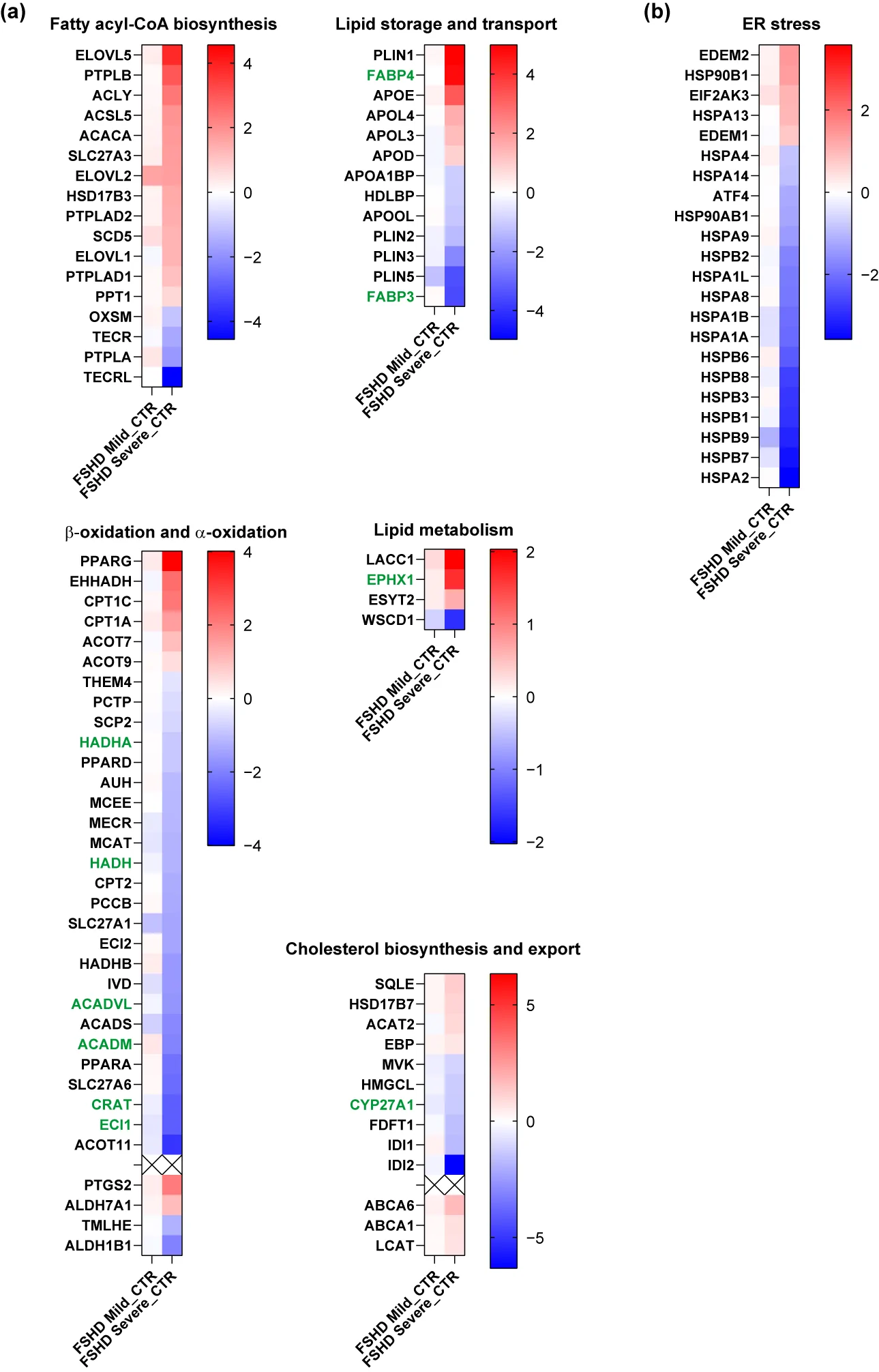
Differential expression of lipid-related pathways in FSHD muscles. Heatmaps show transcript-level changes (RNA-seq) across samples from mild FSHD, severe FSHD, and matched healthy controls (CTR). Gene sets are grouped by pathway: (**a**) fatty acyl-CoA biosynthesis, lipid storage and transport, β- and α-oxidation, general signaling from lipid metabolism, cholesterol biosynthesis and export, and (**b**) ER stress. Color scales represent log-fold changes relative to controls (red = upregulation; blue = downregulation). Genes highlighted in green indicate transcripts corresponding to proteins that were also significantly altered in the parallel proteomics analysis (Figure 1).

We focused our attention on gene expression involved in fatty acyl-CoA biosynthesis, lipid storage and transport, β- and α-fatty acid oxidation, cholesterol biosynthesis and export (Figure 3a) and on ER stress (Figure 3b).

#### 3.1.1. Lipid Metabolism (Figure 3a)

Concerning fatty acyl-CoA biosynthesis, the most highly expressed transcripts in severe samples included elongation of very long-chain fatty acids protein 5 (ELOV5), 3-hydroxyacyl-CoA dehydratase 2, (PTPLB), which catalyzes the dehydration step in long-chain fatty acid elongation, and ATP citrate lyase (ACLY), responsible for cytosolic acetyl-CoA production involved in lipogenesis and cholesterogenesis. Also included was acetyl-CoA carboxylase alpha (ACACA), which synthesizes malonyl-CoA, the rate-limiting intermediate in fatty acid synthesis. This protein expression pattern is compatible with reduced mitochondrial fatty acid import and β-oxidation. In addition, the increased LDHB and decreased LDHA levels previously described [7] may suggest a shift in lactate metabolism toward lactate-to-pyruvate conversion. Taken together, these findings suggest a metabolic shift characterized by reduced mitochondrial fatty acid oxidation and a potential increase in malonyl-CoA availability, which may simultaneously inhibit fatty acid import into mitochondria and favor de novo fatty acid synthesis. Strongly decreased transcripts in patients with severe FSHD included trans-2,3-enoyl-CoA reductase-like (TECRL), 3-hydroxyacyl-CoA dehydratase 1(PTPLA), and trans-2,3-enoyl-CoA reductase (TECR), all involved in very long-chain fatty acid synthesis for sphingolipid production, suggesting a rebalancing rather than a uniform increase in elongation across chain lengths. These transcripts were unchanged or slightly increased in mild cases.

Concerning lipid storage and transport, patients with severe FSHD showed increased levels of perilipin 1 (PLIN1), fatty acid-binding protein 4 (FABP4), APOE, APOL4, APOL3, and APOD. Decreased levels were observed for FABP3 and PLIN5 (slightly decreased also in mild), PLIN3, and PLIN2.

The differential expression analysis revealed significant modulation of genes involved in lipid metabolism, membrane dynamics, and glycan modification. Laccase domain-containing 1 (LACC1), upregulated in patients with severe FSHD, encodes an oxidoreductase involved in fatty-acid oxidation and inflammasome activation, suggesting enhanced lipid catabolism coupled with inflammatory signaling. Epoxide hydrolase 1 (EPHX1), also upregulated, plays a key role in the metabolism of endogenous lipids, particularly epoxide-containing fatty acids, indicating increased processing of bioactive lipid intermediates. Similarly, extended synaptotagmin-2 (ESYT2) was upregulated and is known to tether the endoplasmic reticulum to the plasma membrane, facilitating the formation of membrane contact sites and contributing to intracellular lipid transport and membrane lipid homeostasis. In contrast, WSC domain containing 1 (WSCD1), encoding a sialate O-sulfotransferase involved in glycolipid modification (e.g., GM1 ganglioside sulfation), was downregulated. The WSCD1 downregulation suggests disruption of lipid-raft organization and of membrane microdomains.

Cholesterol biosynthesis-related genes, including squalene epoxidase (SQLE), 17-beta-hydroxysteroid dehydrogenase 7 (HSD17B7), acetyl-CoA acetyltransferase 2 (ACAT2), and emopamil-binding protein (EBP), were modestly increased in patients with severe FSHD, suggesting a transcriptional trend toward enhanced cholesterol biosynthesis and/or processing. Conversely, isopentenyl-diphosphate delta isomerase 2 (IDI2), a key precursor for isoprenoid synthesis, was strongly decreased. This decrease may indicate reduced IPP/DMAPP availability, which could impair prenylation-dependent processes—potentially affecting localization and signaling of prenyl-dependent regulators such as small GTPases.

Concerning cholesterol trafficking, slightly increased ABCA6 was observed in patients with severe FSHD. ABCA1, which participates in phospholipid transfer to apolipoproteins during nascent HDL formation, and LCAT were also modestly increased. Collectively, these expression changes are consistent with enhanced cholesterol efflux and/or HDL-related processing, suggesting altered cholesterol handling rather than straightforward accumulation.

Transcripts from enzymes involved in mitochondrial fatty acid β-oxidation were decreased, including acyl-CoA thioesterase 11 (ACOT11), carnitine O-acetyltransferase (CRAT), carnitine palmitoyltransferase 2 (CPT2), and enoyl-CoA delta isomerase 1 (ECI1), a key mitochondrial enzyme involved in beta-oxidation of unsaturated fatty acids, suggesting a reduced mitochondrial fatty acid oxidation capacity. In contrast, transcripts of enzymes of the β-oxidation pathway, including peroxisome proliferator-activated receptor gamma (PPARG), enoyl-CoA hydratase and 3-hydroxyacyl CoA dehydrogenase (EHHADH) were increased, indicating a compensatory or preferential peroxisomal oxidation route for long-chain/very-long-chain substrates. Concerning fatty acid α-oxidation, genes showed fewer alterations, suggesting a lesser impact of this pathway in severe disease.

Severe FSHD appears to feature coordinated lipid metabolic remodeling, including enhanced cytosolic lipid synthesis and elongation, rebalanced lipid storage and transport, modestly increased cholesterol handling, and a metabolic shift away from mitochondrial FA oxidation toward peroxisomal pathways. This could reflect an adaptive response to lipid overload, inflammation, and increased energy demands in severe disease.

#### 3.1.2. Stress Response (Figure 3b)

Many genes involved in stress response were at variance in patients with severe FSHD. Increased levels were observed for ER degradation enhancing alpha-mannosidase-like protein1 and 2 (EDEM1, EDEM2), involved in the ER-associated degradation (ERAD) pathway targeting misfolded glycoproteins for degradation, heat shock protein 90 beta family member 1 (HSP90B1), heat shock protein family A member 13 (HSPA13), and eukaryotic translation initiation factor 2-alpha kinase 3 (EIF2AK3/PERK), all associated with ER stress signaling.

Conversely, transcripts of several heat shock proteins (HSPA2, HSPB9, HSPB8, HSPB1) were markedly downregulated. These proteins are involved in protein refolding and cooperate with Bcl-2-associated athanogene 3 (Bag3) to stimulate macroautophagy, suggesting a reduced capacity for chaperone-mediated refolding and a potentially impaired macroautophagic response. This could shift proteostasis toward degradation over refolding but also limit clearance via autophagy, confirming results from our previous study [7].

Transcripts from muscle-related chaperones, including heat shock protein family B members 6, 7, and 3 (HSPB6, HSPB7, HSPB3), which influence muscle relaxation and actin dynamics, were also decreased, suggesting an increase in unfolded or stress-damaged proteins impacting the actin cytoskeleton and muscle contraction that may affect cytoskeletal maintenance and muscle function.

#### 3.1.3. Inflammation and Immune Response

**Figure 4:**
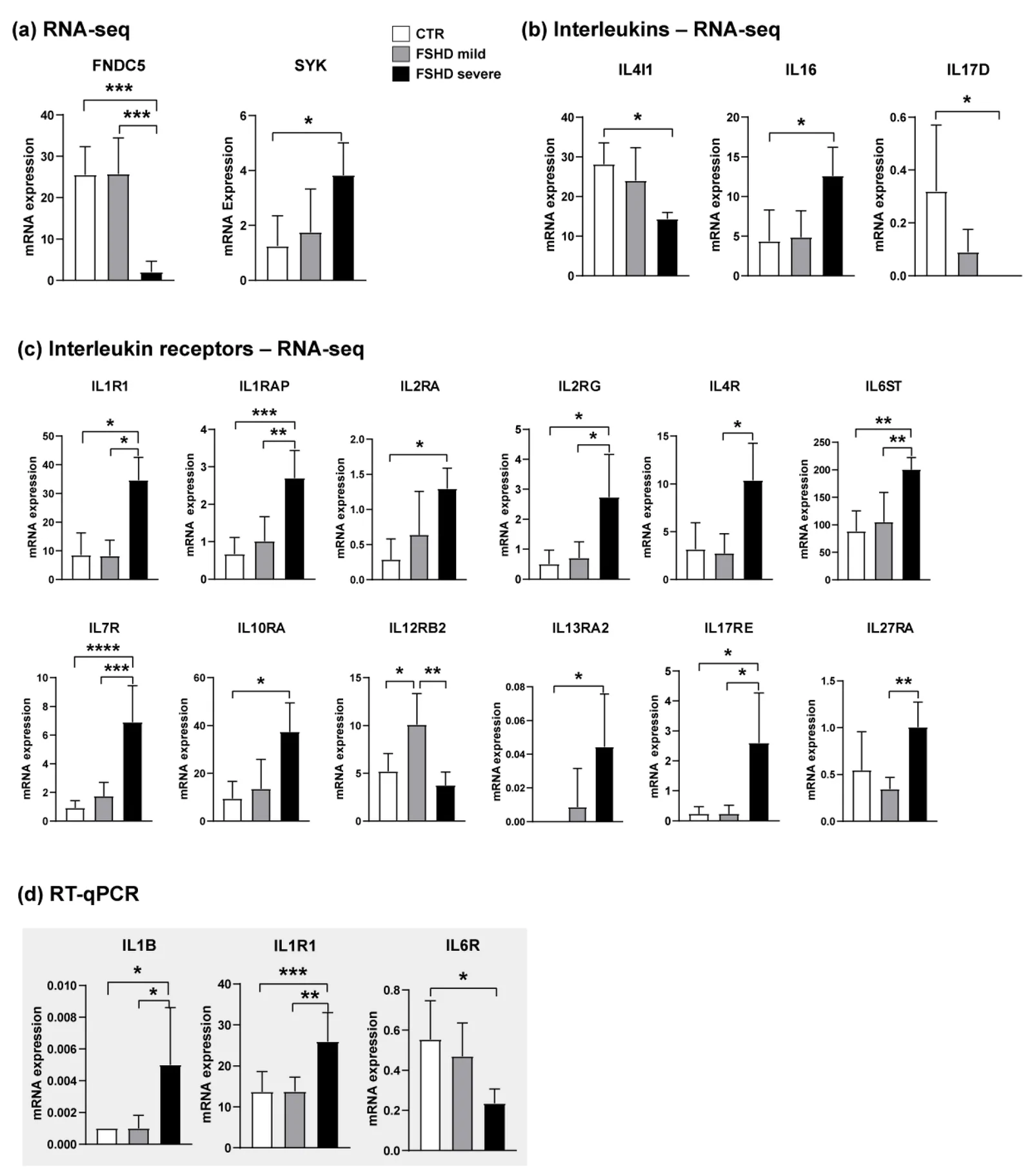
RNA-seq analysis of selected genes involved in inflammation and immune signaling in skeletal muscle from control subjects and patients with mild and severe facioscapulohumeral muscular dystrophy (FSHD). (**a**) RNA-seq analysis of FNDC5 and SYK mRNA expression. (**b**) RNA-seq analysis of differentially expressed interleukins. (**c**) RNA-seq analysis of differentially expressed interleukin receptors. (**d**) RT-qPCR analysis of selected inflammatory genes (IL1B, IL1R1, and IL6R). White bars represent control subjects (CTR), light grey bars represent mild FSHD patients, and black bars represent severe FSHD patients. Data are presented as mean ± SD. Statistical significance is indicated as follows: * *p* < 0.05; ** *p* < 0.01; *** *p* < 0.001; **** *p* < 0.0001 (one-way ANOVA followed by Tukey’s post hoc test).

The release of hyaluronic acid in muscle tissue, together with the dysregulation of heat shock proteins observed in our previous study [7] and confirmed by RNA-seq analysis, prolongs the activation and recruitment of immune cells, inducing a chronic inflammatory state in severe FSHD, as indicated by IPA canonical pathway results. RNA-seq data indicated decreased levels in patients with severe FSHD, not observed in mild patients, of fibronectin type III domain-containing 5 (FNDC5, irisin), a protective molecule involved in AMPK signaling and autophagy regulation, and increased levels of spleen tyrosine kinase (SYK)-associated reactive oxygen species (ROS) and inflammatory signaling pathways (Figure 4a).

**Figure 5:**
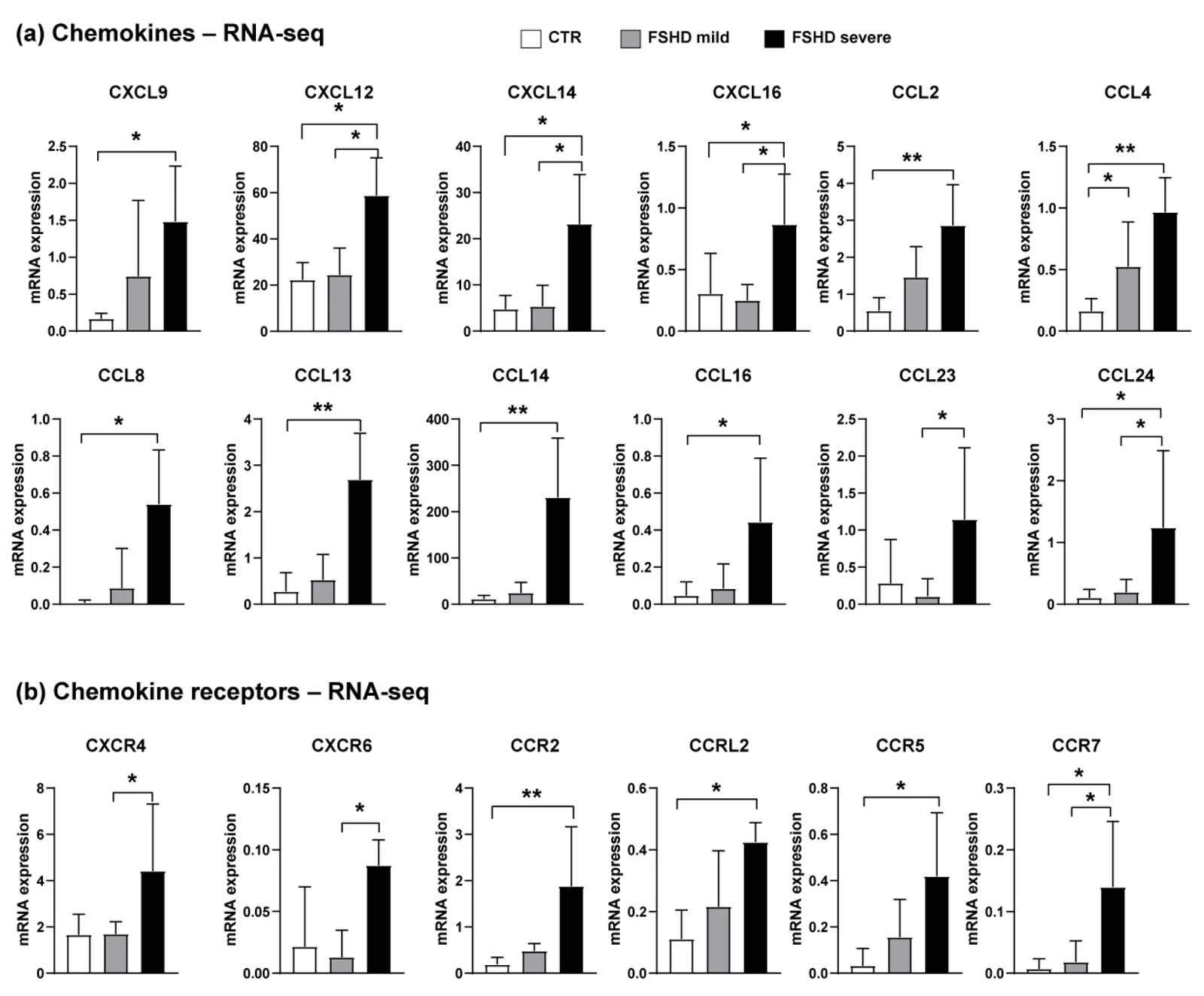
RNA-seq analysis of selected genes involved in inflammation and immune signaling in skeletal muscle from control subjects and patients with mild and severe facioscapulohumeral muscular dystrophy (FSHD). (**a**) RNA-seq analysis of differentially expressed chemokines. (**b**) RNA-seq analysis of differentially expressed chemokine receptors. White bars represent control subjects (CTR), light grey bars represent mild FSHD patients, and black bars represent severe FSHD patients. Data are presented as mean ± SD. Statistical significance is indicated as follows: * *p* < 0.05; ** *p* < 0.01 (one-way ANOVA followed by Tukey’s post hoc test).

Increased SYK expression in patients with severe FSHD, not observed in mild FSHD, is consistent with activation of SYK-mediated inflammatory signaling. This response is accompanied by broad remodeling of cytokine signaling, characterized by marked upregulation of several interleukin receptors (Figure 4b–d) together with increased expression of multiple chemokines and chemokine receptors (Figure 5). Collectively, these data reveal extensive remodeling of inflammatory and immune signaling in severe FSHD, characterized by altered expression of selected interleukins, marked upregulation of interleukin receptors, and increased expression of multiple chemokine ligands and receptors. These findings support enhanced immune cell recruitment and inflammatory signaling, consistent with the IPA results and our previous proteomic observations [7].

#### 3.1.4. Sphingolipid Signaling

**Figure 6:**
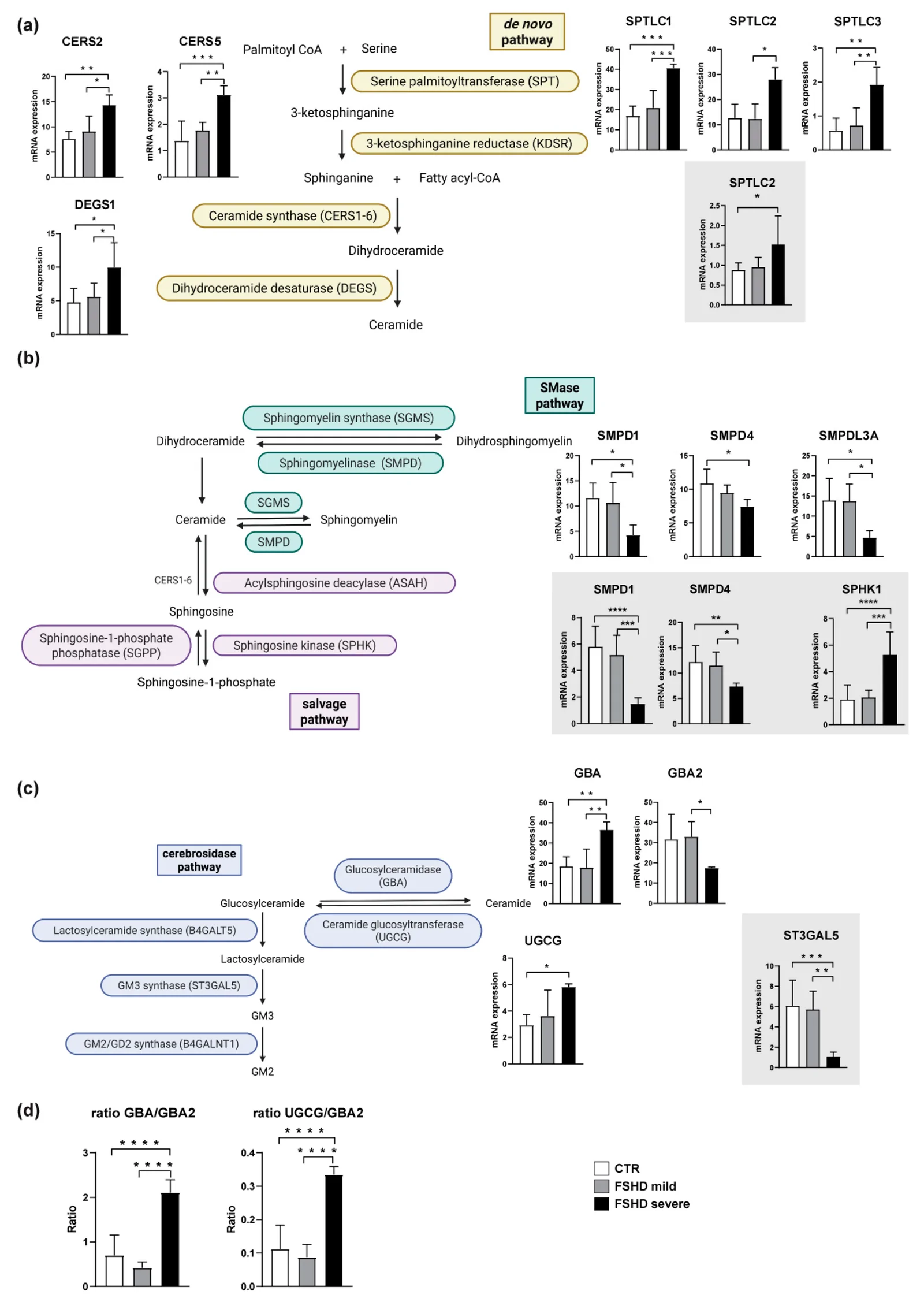
Overview of the main sphingolipid metabolic pathways and expression of the corresponding enzymes in skeletal muscle from control subjects and patients with mild and severe facioscapulohumeral muscular dystrophy (FSHD). (**a**) De novo sphingolipid synthesis pathway. (**b**) Sphingomyelinase and salvage pathways. (**c**) Cerebrosidase pathway. Bar graphs show mRNA expression levels determined by RNA-seq for the enzymes involved in each pathway. Grey boxed panels indicate targeted RT-qPCR analysis of selected differentially expressed genes. (**d**) Ratios of GBA/GBA2 and UGCG/GBA2 mRNA expression. White bars represent control subjects, light grey bars represent mild FSHD patients, and black bars represent severe FSHD patients. Data are presented as mean ± SD. Statistical analysis was performed using one-way ANOVA followed by Tukey’s post hoc test (* *p* < 0.05; ** *p* < 0.01; *** *p* < 0.001; **** *p* < 0.0001).

ER stress, lipotoxicity, and inflammation are known to affect lipid raft organization and sphingolipid composition and signaling. To get deeper into this relationship in muscle tissue, transcripts from sphingolipid enzymes were analyzed to monitor sphingolipid metabolism, including the de novo synthesis pathway, the sphingomyelinase (SMase) and salvage pathway, and the cerebrosidase pathway (Figure 6 and Supporting Information Figure S2).

RNA-seq data for enzymes involved in the de novo sphingolipid synthesis are shown in Figure 6a and Supporting Information Figure S2a. Increased levels of all isoforms of serine palmitoyltransferase long chain (SPTLC1, SPTLC2, SPTLC3), the rate-limiting enzyme in sphingolipid biosynthesis, were observed in patients with severe FSHD. Targeted RT-qPCR confirmed SPTLC2 increase. The second enzyme in the pathway, leading to sphinganine and fatty acyl-CoA, 3-ketodihydrosphingosine reductase (KDSR), increased in patients with severe FSHD, although not significantly, suggesting its contribution to ceramide synthesis. Concerning ceramide synthases, increased levels of ceramide synthase 2 and 5 (CERS2, CERS5) were observed in patients with severe FSHD compared to CTR and mild FSHD. In contrast, a tendency to increase, although not statistically significant, was observed for ceramide synthase 4 (CERS4), whereas CERS6 levels were unchanged. Dihydroceramide desaturase 1 (DEGS1), catalyzing the final step in de novo ceramide synthesis, was increased in severe cases.

Figure 6b and Supporting Information Figure S2b show RNA-seq results for enzymes involved in the SMase and salvage pathway. A tendency toward increased levels of enzymes promoting sphingomyelin synthesis (sphingomyelin synthase 1 and 2, SGMS1 and SGMS2) was observed. Enzymes promoting the reverse reaction leading to the synthesis of ceramide from sphingomyelin (sphingomyelin phosphodiesterase SMPD1, SMPD4) and SMPDL3A, a lipid/ceramide-related enzyme anchored to the cell membrane with a sphingomyelinase-like activity that can affect the balance of sphingomyelin and ceramide, were decreased in patients with severe FSHD. The decreased expression of SMPD1 and SMPD4 was further confirmed by RT-qPCR. Furthermore, RNA-seq data indicates no significant changes in N-acylsphingosine amidohydrolase (ASAH). Targeted RT-qPCR analysis showed increased SPHK1 expression in patients with severe FSHD only.

Figure 6c and Supporting Information Figure S2c show the transcript levels of enzymes involved in the cerebroside pathway. RNA-seq data indicate, increased levels of glucosylceramidase beta (GBA) and of UDP-glucose ceramide glucosyltransferase (UGCG) in patients with severe FSHD, suggesting activation of synthesis of hexosylceramide (HexCer) that can be converted to ceramide (Cer) by GBA or to lactosylceramide (LacCer) by beta-1,4-galactosyltransferase 5 (B4GALT5). B4GALT5, ST3 beta-galactoside alpha-2,3-sialyltransferase 5 (ST3GAL5), and beta-1,4-N-acetyl-galactosaminyltransferase 1 (B4GALNT1), which regulate globoside synthesis, showed a tendency to decrease. This is paralleled by decreased levels of WSCD1, a sialilate O-sulfotransferase involved in GM1 ganglioside sulfation. ST3GAL5 by RT-qPCR was significantly decreased in patients with severe FSHD, suggesting that sialylation is impaired and the flux toward LacCer synthesis is reduced.

A closer look at patients with mild FSHD reveals a deviation in the globoside pathway. When data are ordered by disease score, the node is represented by levels of UGCG and GBA, suggesting that mild cases may be more prone to synthesize HexCer. GBA and GBA2, lysosomal and non-lysosomal enzymes, respectively, catalyze the hydrolysis of HexCer into glucose and ceramide. In humans, mutations that reduce beta-glucocerebrosidase activity cause Gaucher disease (GD), a rare lysosomal storage disorder with neurological involvement and Parkinsonism-like symptoms [32]. Previous work from our group and others indicates that several lipid species, mono- and dihexosylceramides (HexCer, GalCer, LacCer), as well as Cer, dihydroceramide (dhCer), sphingosine, and sphingomyelin (SM), exhibit a pronounced imbalance in animal models of neurodegeneration and in Parkinson’s disease patients, contributing to neurodegenerative decline [33].

In patients with mild FSHD, the cerebrosidase pathway does not appear inhibited; GBA and GBA2 transcript levels are comparable to controls, and a non-significant increase in B4GALT5, ST3GAL5, and B4GALNT1 was observed. The ratios GBA/GBA2 and UGCG and GBA2 (Figure 6d) indicates unbalance toward GBA2 in severe patients. Reduced GBA activity in lysosomes and leakage of its substrates outside the lysosomes might lead to an increased generation of ceramide and sphingosine at the ER and cis-Golgi. Ceramide can be further broken down to sphingosine and fatty acid by neutral ceramidases. Sphingosine is the cytotoxic metabolite that causes some of the defects associated with Gaucher disease, at least upon its chronic accumulation [34].

In patients with severe FSHD, there is support for upregulated de novo ceramide/sphingolipid synthesis, indicated by higher expression of SPTLC1/2/3, KDSR, CERS2/CERS5, and DEGS1, collectively steering toward ceramide generation via the de novo pathway. Additionally, signals from SGMS1/2, ASAH1, and SGPP1, though not statistically significant, suggest sphingomyelin synthesis and recycling of sphingosine into long-chain ceramides. This shift could elevate ceramide pools, a change associated with lipotoxicity and inflammatory signaling in muscle, confirming results from our previous study [7]. The cerebrosidase pathway appears inhibited, with decreased levels of ST3GAL5 (Figure 6c).

#### 3.1.5. ER Stress and EVs Generation

Collectively, these results suggest an increase in the expression of enzymes promoting ceramide synthesis, sphingosine accumulation, and reduced conversion of sphingmyelin to ceramide, consistent with decreased SMPD levels and increased levels of the calcium-binding protein SYT1. Based on these observations, we hypothesize that metabolic dysregulation, lipotoxicity, and endoplasmic reticulum (ER) stress converge to activate signaling pathways involving sphingolipids and extracellular vesicles (EVs) production.

**Figure 7:**
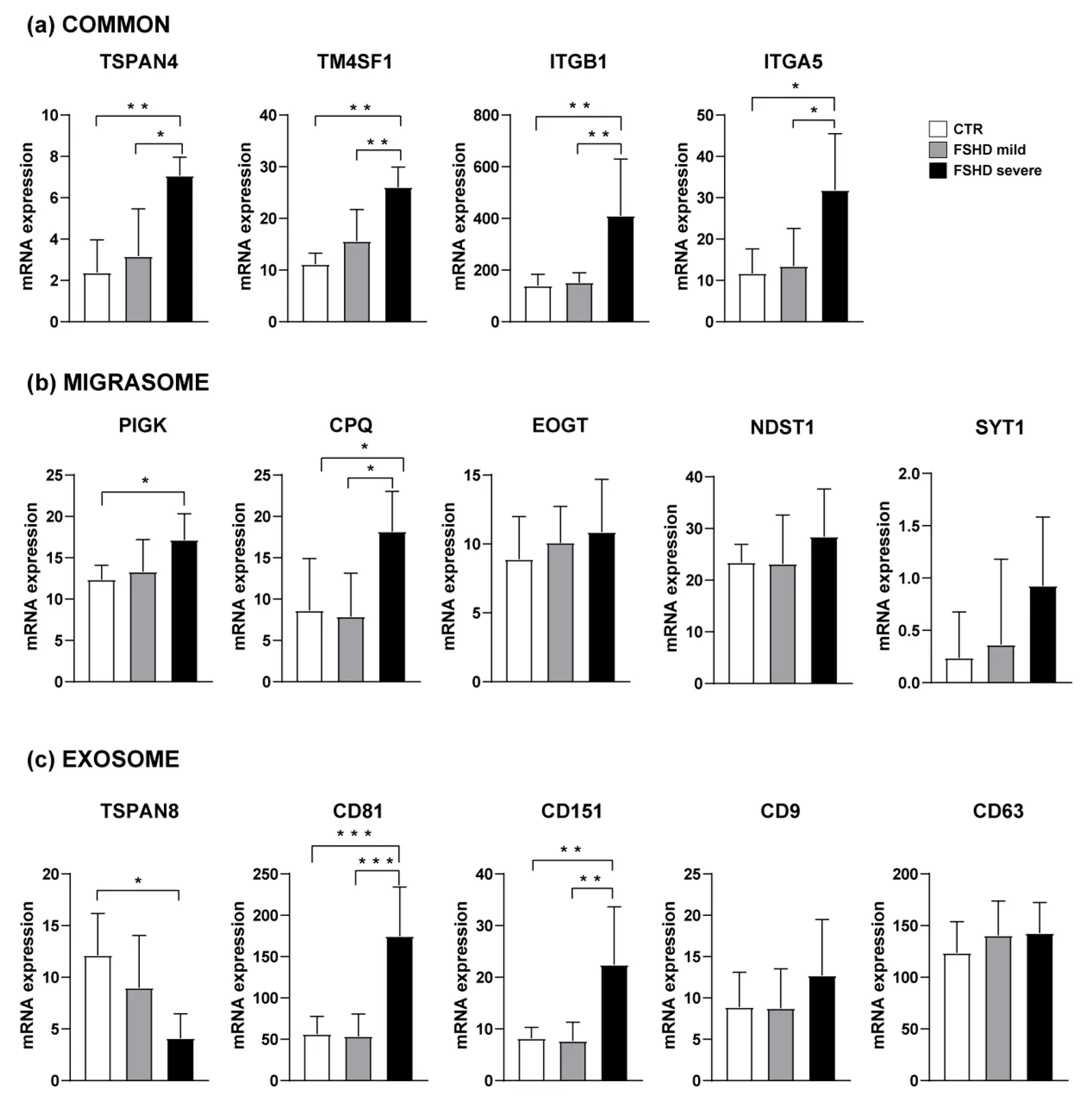
RNA-seq analysis of migrasome- and exosome-associated marker genes in skeletal muscle from control subjects and patients with mild and severe facioscapulohumeral muscular dystrophy (FSHD). (**a**) mRNA expression of genes commonly associated with migrasomes and exosomes (TSPAN4, TM4SF1, ITGB1, and ITGA5). (**b**) mRNA expression of migrasome-associated marker genes (PIGK, CPQ, EOGT, NDST1, and SYT1). (**c**) mRNA expression of exosome-associated marker genes (TSPAN8, CD81, CD151, CD9, and CD63). White bars represent control subjects (CTR), light grey bars represent mild FSHD patients, and black bars represent severe FSHD patients. Data are presented as mean ± SD. Statistical significance is indicated as follows: * *p* < 0.05, ** *p* < 0.01, *** *p* < 0.001 (one-way ANOVA followed by Tukey’s post hoc test).

RNA-seq analysis revealed increased expression of the common migrasome/exosome markers TSPAN4, TM4SF1, ITGA5, and ITGB1, together with the migrasome-associated genes CPQ, PIGK, SYT1, and the exosome-associated markers CD81 and CD151, predominantly in patients with severe FSHD (Figure 7). This transcriptional signature is consistent with enhanced extracellular vesicle biogenesis in severe disease.

These molecular findings correlate with ultrastructural alterations observed by transmission electron microscopy (TEM) in FSHD patients, including accumulation of granular material in intermyofibrillar and subsarcolemmal regions and disruption of contractile elements, generating abnormal myofibrillar organization [29].

Collectively, these findings suggest that lipotoxicity-driven EV formation represents a previously underappreciated mechanism contributing to sphingolipid redistribution, structural muscle damage, and altered intercellular communication in severe disease conditions.

### 3.2. Sphingolipidomic Analysis of Serum Samples

The rationale for addressing this issue was to address questions arising from our RNA-seq findings on the expression levels of enzymes involved in the sphingolipid pathway. Specifically, we examined whether ceramide and its derivatives, together with markers of EV biogenesis, are detectable in serum. Based on these results obtained in muscle samples, we expected that increased levels of enzymes involved in ceramide synthesis and sphingomyelin metabolism, SGMS1, SGMS2, ASAH1, and SGPP1, together with the ratio of UGCG/GBA2 and GBA/GBA2, could be associated with changes in the levels of ceramides and globosyl derivatives released in serum. These findings suggest that Cer, HexCer, LacCer, and GM3 could serve as potential serological markers of FSHD severity.

**Figure 8:**
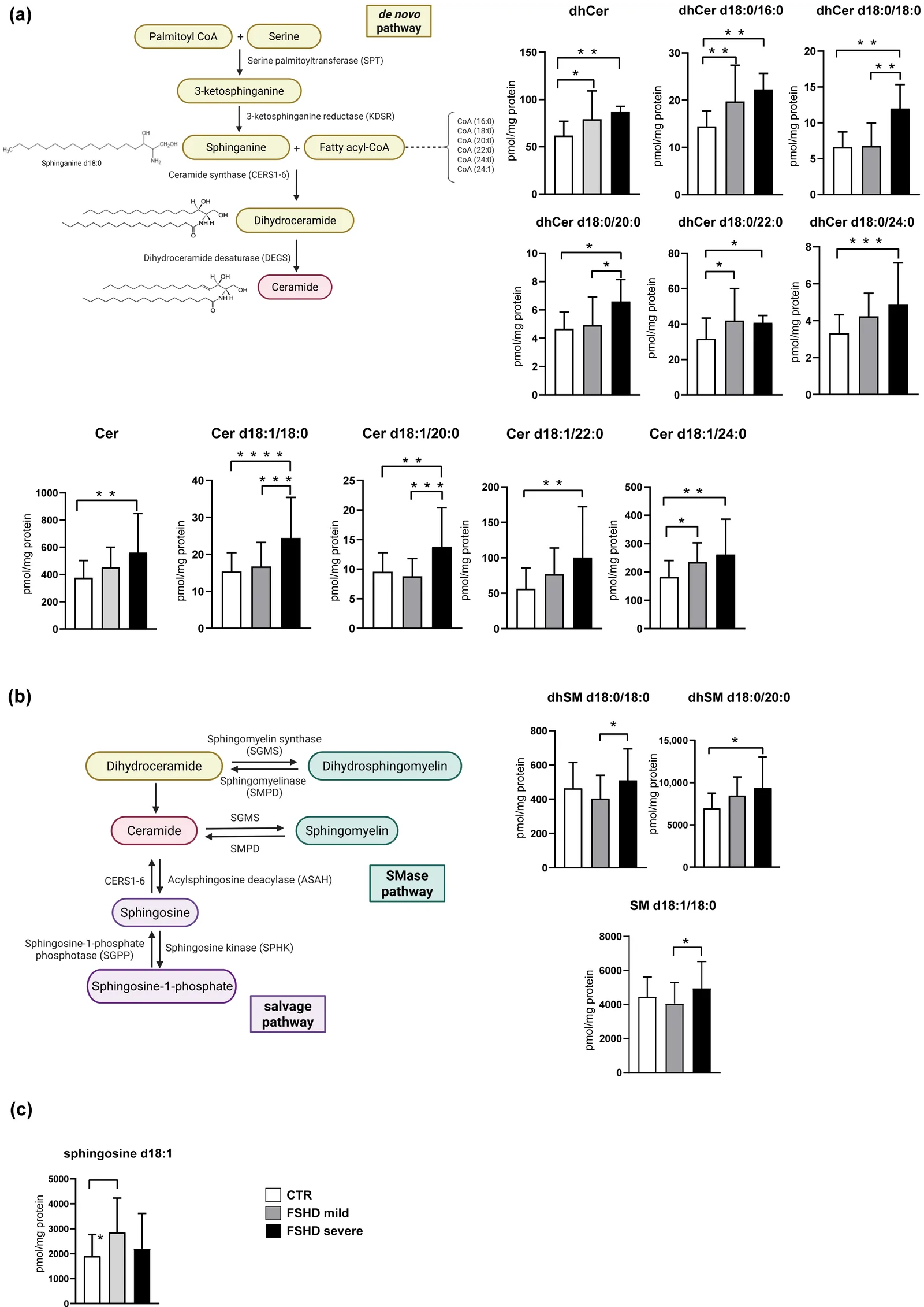
Alterations in sphingolipid species along the de novo, sphingomyelinase (SMase), and salvage pathways in serum from control subjects and patients with mild and severe facioscapulohumeral muscular dystrophy (FSHD). (**a**) De novo sphingolipid synthesis pathway and LC-MS quantification of dihydroceramide (dhCer) and ceramide (Cer) species. (**b**) Sphingomyelinase (SMase) pathway and LC-MS quantification of dihydrosphingomyelin (dhSM) and sphingomyelin (SM) species. (**c**) Salvage pathway and LC-MS quantification of sphingosine and sphingosine-1-phosphate (S1P). White bars represent control subjects, light grey bars represent mild FSHD patients, and black bars represent severe FSHD patients. Lipid species were quantified by LC-MS using internal standards, as described in the Materials and Methods. Data are presented as mean ± SD. Statistical significance is indicated as follows: * *p* < 0.05, ** *p* < 0.01, *** *p* < 0.001, **** *p* < 0.0001 (one-way ANOVA followed by Tukey’s post hoc test or Friedman test followed by Dunn’s post hoc test, as appropriate). The coefficient of variation (CV) was <10%.

Serum samples from mild (*n* = 9) and patients with severe FSHD (*n* = 13) were analyzed and compared with control samples (*n* = 10) using both targeted and untargeted LC-MS/MS approaches. Results from the de novo synthesis pathway are shown in Figure 8a and Supporting Information Figure S3a. Panel 8a shows that total levels of dhCer increased in both mild and severe FSHD patients compared to CTR. Furthermore, changes in levels of specific dhCer chains were observed. Increased levels of dhCer d18:0/16:0 and dhCer d18:0/22:0 were detected in both mild and severe FSHD patients, while dhCer d18:0/18:0, dhCer d18:0/20:0, and dhCer d18:0/24:0 significantly increased in patients with severe FSHD only. No significant changes were observed in dhCer d18:0/24:1.

Concerning ceramide species, total Cer levels significantly increased only in patients with severe FSHD. Specifically, levels of Cer d18:1/18:0 and Cer d18:1/20:0 increased in patients with severe FSHD compared to both controls and mild patients; Cer d18:1/22:0 increased in patients with severe FSHD versus CTR only; Cer d18:1/24:0 increased in mild and severe FSHD patients compared to controls, although the increase was more significant in patients with severe FSHD. No statistically significant changes were observed for Cer d18:1/16:0, Cer d18:1/24:1, and Cer d18:0/26:0.

The Smase and salvage pathways are shown in Figure 8b and Supporting Information Figure S3b. Total dhSM levels showed a slight but not statistically significant increase in patients with severe FSHD compared with CTR. Among the individual species, dhSM d18:0/20:0 was increased in patients with severe FSHD.

Concerning SM, total levels remained unchanged in both mild and severe FSHD patients. Among the individual molecular species, SM d:18:1/18:0 was significantly increased in patients with severe FSHD compared with patients with mild FSHD only, while no significant changes were observed for SM d18:1/16:0, d18:1/20:0, d18:1/22, d18:1/24:0, d18:1/24:1, and d18:1/26:0.

Sphingosine (Sph) d18:1 level was increased in patients with mild FSHD compared to controls only, whereas S1P d18:1 was unchanged in both patients with mild and severe FSHD compared to controls (Figure 8c and Supporting Information Figure S3c).

**Figure 9:**
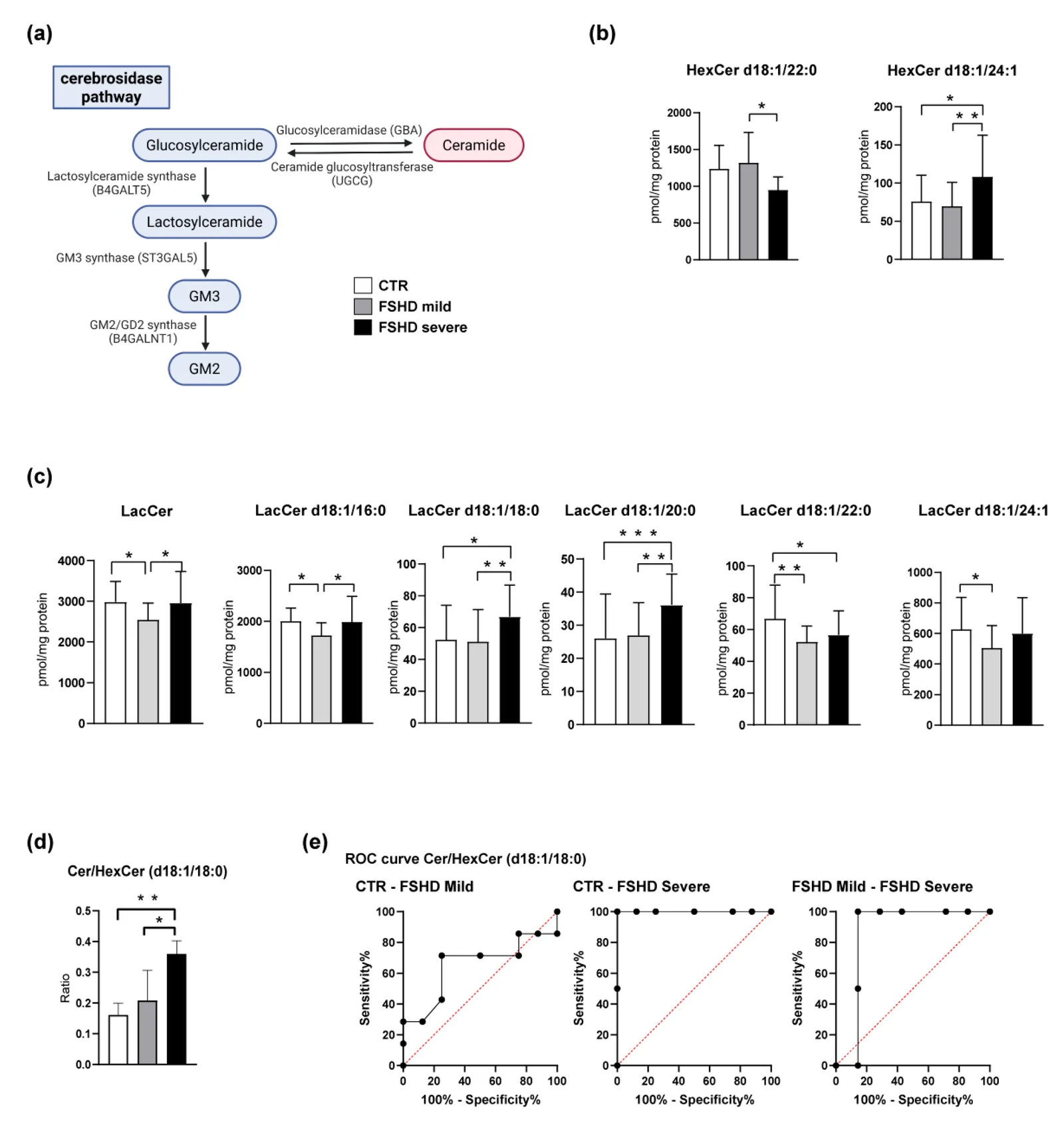
Alterations in sphingolipid species of the cerebrosidase pathway in serum from control subjects and patients with mild and severe facioscapulohumeral muscular dystrophy (FSHD). (**a**) Schematic representation of the cerebrosidase pathway. (**b**) LC-MS quantification of hexosylceramide (HexCer) species. (**c**) LC-MS quantification of lactosylceramide (LacCer) species. (**d**) Ratio of Cer/HexCer (d18:1/18:0). White bars represent control subjects, light grey bars represent mild FSHD patients, and black bars represent severe FSHD patients. Lipid species were quantified by LC-MS using internal standards, as described in the Materials and Methods. Data are presented as mean ± SD. Statistical significance is indicated as follows: * *p* < 0.05, ** *p* < 0.01, *** *p* < 0.001 (one-way ANOVA followed by Tukey’s post hoc test or Friedman test followed by Dunn’s post hoc test, as appropriate). The coefficient of variation (CV) was <10%. (**e**) ROC curves were generated to assess the ability of the Cer/HexCer (d18:1/18:0) ratio to discriminate healthy controls from patients with mild FSHD, healthy controls from patients with severe FSHD, and patients with mild FSHD from those with severe FSHD. The corresponding areas under the curve (AUCs) were 0.652, 1.000, and 0.857, respectively. The dashed red diagonal line represents the reference line corresponding to random classification (AUC = 0.5).

Figure 9a and Supporting Information Figure S4a show the sphingolipid cerebrosidase pathway. Levels of HexCer (Figure 9b and Figure S4b), LacCer (Figure 9c and Figure S4c), and GM3 (Figure S4d) were assessed in patients with mild and severe FSHD. Total levels of HexCer did not change statistically, although a slight decrease was observed in patients with severe FSHD. By analyzing different HexCer species, statistically significant changes were observed in HexCer d18:1/22:0, which decreased in patients with severe FSHD compared to patients with mild FSHD, and in HexCer d18:1/24:1, which increased in patients with severe FSHD compared to patients with mild FSHD and controls.

Total LacCer was decreased in patients with mild FSHD compared with both CTR and patients with severe FSHD. Regarding specific species dysregulation, LacCer d18:1/16:0 decreased in patients with mild FSHD compared with controls and patients with severe FSHD, and LacCer d18:1/18:0 and d18:1/20:0 increased only in patients with severe FSHD. A decreased level of LacCer d18:1/22:0 was observed in both mild and severe FSHD, while LacCer d18:1/24:1 decreased in mild only. No statistically significant changes were observed in GM3 species, although a general decrease was observed in patients with severe FSHD.

A more detailed analysis of HexCer metabolism suggests that in patients with mild FSHD, UGCG and GBA expression promote the synthesis of ß-glucocerebrosidase that catalyzes the hydrolysis of HexCer to glucose and ceramide in mild [35].

In patients with severe FSHD, these results indicate an increased release of ceramides into the serum, specifically ceramide species synthesized from long-chain fatty acids, consistent with the observed increased levels of mRNA transcripts of CERS2 and CERS5.

The evolution of the disease severity ratio of Cer/HexCer (d18:1/18:0) (Figure 9d) was assessed, indicating an increasing trend from controls to mild to severe. This ratio can be indicative of evolution toward a severe condition. ROC curve analysis revealed a differential discriminatory performance according to FSHD severity (Figure 9e). The Cer/HexCer (d18:1/18:0) ratio showed limited ability to discriminate patients with mild FSHD from healthy controls (AUC = 0.652, 95% CI: 0.348–0.955, *p* = 0.3253). Conversely, complete separation between healthy controls and patients with severe FSHD was observed in the present cohort (AUC = 1.000, *p* = 0.0367). The parameter also showed a relatively high AUC when comparing mild and severe FSHD patients (AUC = 0.857, 95% CI: 0.598–1.000), although this difference did not reach statistical significance (*p* = 0.1432). Given the small number of patients per group, these findings should be considered preliminary and require validation in a larger cohort.

### 3.3. Levels of Specific Molecules Involved in Lipid Droplet Assembly in Serum

**Figure 10:**
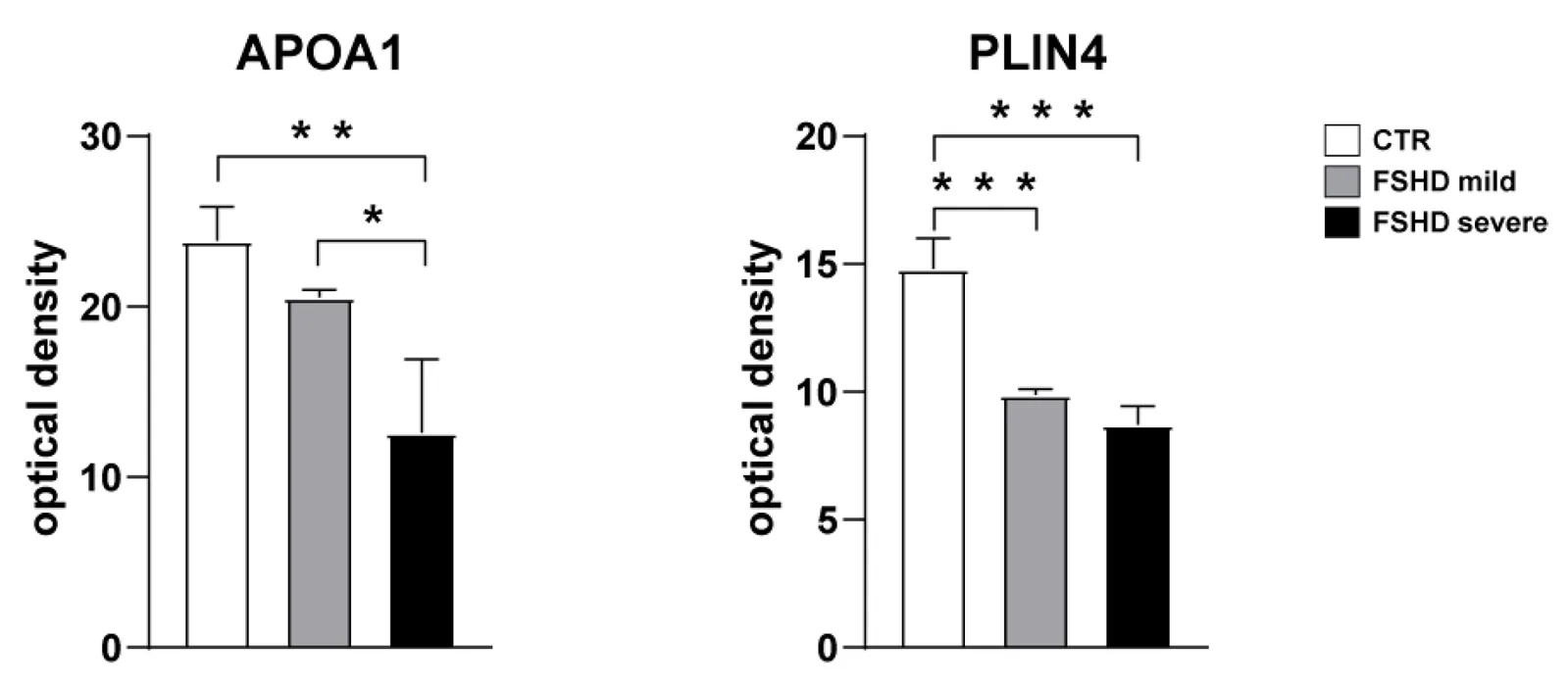
Western blot analysis of APOA1 and PLIN4 protein levels in serum from control subjects and patients with mild and severe facioscapulohumeral muscular dystrophy (FSHD). Densitometric quantification of the immunoreactive bands is shown as optical density. White bars represent control subjects, light grey bars represent mild FSHD patients, and black bars represent severe FSHD patients. Data are presented as mean ± SD. Statistical significance is indicated as follows: * *p* < 0.05; ** *p* < 0.01; *** *p* < 0.001 (one-way ANOVA followed by Tukey’s post hoc test). Full-length blot images are provided in Supporting Information Figure S2.

Results from immunoblotting analysis of serum samples showed an opposite pattern compared with muscle tissue. Decreased APOA1 levels in serum characterized patients with severe FSHD relative to controls and mild cases (Figure 10), opposite to the trend observed in muscle. Reduced serum PLIN4 levels were observed alongside increased PLIN4 in severe patients in muscle, suggesting that PLIN4-associated lipid droplet remodeling may be driven locally in muscle, whereas circulating PLIN4 likely reflects systemic transport, release, and/or clearance.

## 4. Discussion

Reliable serum biomarkers are urgently needed to monitor disease progression and therapeutic response, particularly in a slowly progressive disorder such as FSHD. This need arises for two main reasons: muscle biopsies are no longer ethically justifiable, not all muscles are equally affected, and the response to therapy can have a different time course compared to physiological changes [36,37,38]. Ideally, such biomarkers should be directly involved in, or associated with, muscle impairment.

What do we learn from the muscle proteome and transcriptome in mild versus severe patients? In mild patients, compensatory mechanisms activated to protect against muscle decline are evident at both the proteomic and transcriptomic levels. A key distinguishing feature of patients with severe FSHD is the impairment of fatty acid β-oxidation, leading to the accumulation of long-chain fatty acids that are not efficiently utilized by mitochondria for energy production. These metabolic alterations may, in turn, contribute to activation of the unfolded protein response (UPR), as supported by proteomic and RNA-seq signatures and by increased levels of hyaluronic acid, Caspase-8, Caspase-1, and vesicle-associated membrane protein 2 (VAMP2) [7].

Decreased levels of Irisin and increased levels of SYK were observed. Irisin, as a protective/adaptive signal (PGC-1α/mitochondrial-linked), is often associated with metabolic support, mitochondrial function, and anti-inflammatory effects. In muscle disease, lower Irisin can reflect impaired mitochondrial/endurance programs or reduced compensatory capacity. The negative relationship observed between irisin and SYK in our patients may reflect that increased inflammatory signaling mediated by SYK suppresses adaptive metabolic programs, alongside reduced mitochondrial/adaptive signaling mediated by Irisin [39].

### 4.1. The Impact of Long-Chain Fatty Acid Dysregulation on Sphingolipid Signaling

IPA analysis of the RNA-seq data, together with our previous results, indicated activation of the UPR and ER stress pathway in patients with severe FSHD. Analysis of enzymes involved in ceramide synthesis revealed increased levels of enzymes leading to dhCer production, along with elevated expression of CERS2 and CERS5. This suggests that excess long-chain fatty acids, not utilized for energy production, may be redirected toward the endoplasmic reticulum, where they could be converted into specific ceramide species that may contribute to UPR activation in muscle [40]. Serum sphingolipid results indicate increased levels of CERS5-related dhCer species, such as dhCer d18:0/16:0, which do not contribute to UPR activation. However, increased levels of CERS2 (dhCer d18:0/18:0, dhCer d18:0/20:0, dhCer d18:0/22:0) and CERS4 (dhCer d18:0/24:0) associated species were observed. Concerning ceramide, increased levels of CERS5 (Cer d18:1/18:0, Cer d18:1/20:0) and CERS2 (Cer d18:1/22:0, Cer d18:1/24:0) associated species were observed. Only species promoted by CERS2 are considered bioactive lipids capable of mediating paracrine signaling in response to lipotoxic stress [40].

One proposed mechanism for UPR/ER stress onset is that long-chain ceramides (C18 and longer) have reduced intermembrane mobility, leading to their accumulation in subcellular compartments and potentially triggering apoptosis [41]. Ceramides can further boost their own synthesis by directly inhibiting mitochondrial complex III, increasing reactive oxygen species, and stimulating serine palmitoyltransferase activity [42]. Additionally, ceramides raise mitochondrial oxyradical production by inhibiting Bcl-2 through activation of protein phosphatase 2A [43].

In patients with severe FSHD, a trend toward increased SGMS2 expression, together with decreased SMPDs, may contribute to the accumulation of specific sphingomyelin species (SM d18:1/18:0 and dhSM d18:0/18:0), which, in combination with cholesterol esters and increased levels of SYT1, can promote microvesicle formation and release [44,45,46,47,48]. Further studies are required to precisely define the molecular events associated with this signaling.

In our and other previous studies, the sphingolipid dysregulation in serum has been associated with the decline of muscle mass [16,23,49,50], demonstrating an association between muscle mass loss and sphingolipid signaling. Furthermore, changes in levels of several Cer species in serum were identified in healthy young subjects undergoing prolonged bed rest (180 days). In these subjects, treatment with a cocktail of omega-3 and antioxidants normalized the long-chain ceramide and sphingomyelin species [51], suggesting a potential relationship between decline of muscle mass, sphingolipid signaling, and the role of antioxidants in the recovery of muscle mass and function in healthy subjects. In astronauts on long-duration missions, increased HexCer levels were associated with changes in muscle fiber metabolic phenotype, as well as alterations in contractility and fatigability [52,53].

Moreover, the association of cardiovascular risk with glycerophospholipids, glycerolipids, and the sphingolipidome is supported by several studies [54,55,56], and recently the potential of high-throughput MS lipidomics to determine cardiovascular risk in an Australian population has been demonstrated [57].

Concerning muscular dystrophies, Laurila et al. analyzed a publicly available dataset from muscle biopsies, derived from patients with different muscular dystrophies (including FSHD patients) vs. healthy controls, and evaluated the role of the sphingolipid de novo synthesis pathway by comparing transcript abundance of enzymes of the pathway. Dysregulation of specific ceramide synthases was observed, characterizing different muscle disorders [58]. However, limitations in patient stratification, particularly in FSHD severity, restrict the interpretation of these findings.

In our dataset, analysis of serum from patients with mild FSHD stratified by age and clinical severity suggests that disease progression is associated with progressive alterations in sphingolipid profiles. A key regulatory node is represented by GBA and UGCG levels, which are involved in promoting flux towards globoside synthesis in patients with mild FSHD, whereas this pathway is less active in patients with severe FSHD. Accordingly, the balance between ceramides and HexCer shifts toward ceramide accumulation in patients with severe FSHD, while it appears to decrease or remain balanced in patients with mild FSHD.

In many muscular and neuromuscular disorders, diverse primary defects, such as transporter, lysosomal regulatory, membrane, mitochondrial/autophagy, or trafficking abnormalities, can converge on shared upstream stress pathways. When autophagy–lysosome trafficking is impaired, lysosomal clearance slows and sphingolipid/glycosphingolipid metabolism often becomes dysregulated, which can secondarily alter GBA-related flux through changes in substrate handling, compensatory enzyme regulation, and accumulation of upstream lipids. ER stress, disrupted lipid homeostasis, and chronic inflammation/oxidative stress can further remodel lipid processing and lysosomal biogenesis, shifting key GBA “nodes.” In addition, membrane and trafficking defects can change glucocerebroside delivery and processing, causing GBA pathway signatures to emerge not only in primary lysosomal diseases but also as downstream effects in broader neuromuscular conditions. Consistent with this, GBA-linked pathway involvement has been reported across multiple disorders, including DMD/BMD, LGMD, myotubular myopathy, inclusion body myopathy, ALS, and SMA, supporting the relevance of this pathway convergence point [59].

Furthermore, the role of sphingolipid dysregulation in muscular dystrophies and neuromuscular disorders has mainly been investigated in animal and cell models where specific species levels have been assessed by LC-MS/MS [22,52]. In patients, the amount of muscle required for sphingolipid analysis hampers the direct quantitative assessment in muscle tissue, and studies mainly addressed enzyme transcripts regulating sphingolipid synthesis.

### 4.2. Why We Expected That Sphingolipids Are Released in Blood?

Migrasomes are large vesicular structures generated along retraction fibers during cell migration and are enriched in tetraspanins such as TSPAN4 and TM4SF1, as well as integrins. Their formation is tightly linked to cytoskeletal dynamics and membrane remodeling, which act in the spatial redistribution of cellular components, including lipids and damaged mitochondria, suggesting a functional interplay with mitophagy pathways [60,61,62].

Although the role of migrasomes in skeletal muscle is still poorly defined, recent studies suggest their involvement in muscle regeneration and stress responses. Retraction fibers, which serve as platforms for migrasome biogenesis, are integral to cytoskeletal reorganization during muscle repair. Importantly, a growing body of evidence highlights a functional link between EV-mediated pathways and intracellular degradation systems. Both exosomes and migrasomes need sphingomyelin, ceramide, and cholesterol for their assembly [46]. While exosomes primarily mediate communication, migrasomes can be an alternative route for disposal of damaged cellular components, particularly under conditions of impaired autophagy as suggested by our data. Defects in autophagy and mitophagy could potentially favor the activation of migrasome-dependent clearance mechanisms, thereby contributing to disease progression.

EVs generated in muscle tissue to dispose of damaged material may be released into the blood. Increased levels of SGMS2 observed in patients with severe FSHD may be involved in EV formation, including small exosomes or larger migrasome vesicles [63]. SGMS2 remains localized on retraction fibers. Retraction fibers are involved in the reorganization of the cytoskeleton and the re-establishment of cellular architecture in the regenerating fibers. Retraction fibers are essential components of skeletal muscle cells, contributing to their structure, function, and response to various physiological and pathological conditions [64]. They are distinct from the contractile filaments but work together to enable muscle movement and maintain tissue integrity, serving as the designated site for migrasome formation. Ceramide undergoes conversion to SM mediated by SGMS2, which may contribute to the growth phase of migrasome/exosome formation. Furthermore, both CERS2 and CERS5, essential for long-chain ceramide synthesis, are necessary for migrasome/exosome formation. Increased levels of mRNA transcripts of TM4SF1, TSPAN4, ITGA5, PIGK, and SYK, markers associated with migrasome/exosome signaling [46,47,48], are seen only in severe cases. Migrasome formation could potentially be associated with granular material accumulation in the intermyofibrillar and subsarcolemmal regions, resulting in breaks in contractile material and abnormal myofibril formation observed by TEM in FSHD patients [29].

Another point is the absence of mitophagy and autophagy; the latter was demonstrated in our previous work [7] and is now supported by mRNA transcript analysis indicating repressed mitophagy. This observation further supports the hypothesis that GBA pathway signatures may not simply reflect downstream effects and could be consistent with a possible involvement of migrasomes, which can contribute to the disposal of damaged mitochondria through their processing and excretion from migrating cells [61,65].

PIGK and TM4SF1, specific markers associated with migrasomes, were analyzed in serum samples by immunoblotting. Unfortunately, the direct detection failed, suggesting that specific enrichment of exosomes and migrasomes is required and new enrollment of patients and controls for serum sample collection and EV isolation is ongoing.

However, these results open the possibility to target de novo synthesis of ceramide through inhibition of serine palmitoyltransferase (SPTLC1-3), using myriocin or its derivatives, and to sustain globoside levels [66]. Myriocin and myriocin derivatives are currently not approved for clinical use, even though recent studies on animal models of sarcopenia and the Mdx mouse model have demonstrated their efficacy [67].

Gangliosides (including GM1) contribute to the organization of membrane rafts; sulfation can modulate raft stability and protein partitioning. So, the WSCD1 decrease observed in patients with severe FSHD may lead to an impaired signaling platform. The above-described results may suggest that this signaling can be reversed by targeting globoside levels through the supplementation of modified GM1 (GM1-OS) in conjunction with antioxidant treatments. GM1-OS is characterized by an oligosaccharide component and is marketed as a supplement/drug formulation derived from GM1 aimed at restoring globoside levels. In patients with severe FSHD, GM1-OS could be considered a potential therapeutic approach to counteract muscle deterioration [66,68]. To our knowledge, targeting ceramide and globoside levels in FSHD has not previously been proposed. However, the metabolic node remains the reductive TCA cycle and the absence of β-oxidation, which promotes the accumulation of toxic long-chain fatty acids not utilized for energy production, suggesting that supplementation of GM1-OS [66,68] in conjunction with antioxidant treatment could be explored as a potential therapy to ameliorate dysmetabolism and restore ROS balance in patients with severe FSHD [29,69].

It has to be proven that sustaining globoside levels in conjunction with treatment with an antioxidant cocktail [29,69] can keep disease severity under control.

Nevertheless, sphingolipids can be considered candidate biomarkers for patient follow-up and therapeutic monitoring.

It is of paramount importance that markers have robust methodologies to facilitate their translation in diagnostics. Analytical methodologies for sphingolipid quantification, including extraction protocols, internal standards, and targeted MS approaches, are taken into consideration by the clinical chemistry community and pave the way for a direct translation of sphingolipid monitoring in clinical laboratories [70,71]. Integration of sphingolipid profiling with imaging techniques such as MRI could be a step forward in patient follow-up and clinical trial monitoring.

### 4.3. Limitations of This Study

This is an exploratory study conducted on a small cohort of patients, and the authors are aware that some limitations exist: The sample size did not allow an adequate subgroup power analysis. Although selected RNA-seq findings were validated at the transcript level by RT-qPCR, an independent validation cohort is lacking in this study. Given the relatively small cohort size and the exploratory nature of the study, the transcriptomic findings should be considered hypothesis-generating and interpreted with caution. Further validation in larger, independent cohorts will be required to confirm the reproducibility and generalizability of these findings. An extensive validation is in progress, including the recruitment of a larger number of patients. Further validation of our findings will require integrated proteo-lipidomic approaches, including isolation and characterization of extracellular vesicles from serum followed by targeted and untargeted LC–MS/MS analyses.

Another limitation is represented by a lack of deeper analysis concerning the involvement of fibroadipogenic progenitor cells and their association with mitochondrial dysfunction in FSHD, which has been described by Hengqist et al. [72]. In our dataset, genes related to mitochondrial dysfunction and FAP population expansion were at variance in patients with severe FSHD. However, given the predominantly exploratory nature of this study, we were unable to go deeper into the fibroadipogenic precursor subsets showing dysregulation at RNA-seq. A more quantitative assessment with suitable statistical robustness would have required a larger, more consistent sample size in order to confirm and better quantify the observed differences, given the exploratory nature of this study. These studies are currently in progress.

## Data Availability

The original contributions presented in this study are included in the article/Supplementary Material. Further inquiries can be directed to the corresponding author.

## Supplementary Materials

The following supporting information can be downloaded at https://www.mdpi.com/article/10.3390/antiox15091166/s1: Figure S1: Ingenuity Pathway Analysis of canonical pathways in RNA-seq data. Figure S2: Overview of the main sphingolipid enzymes in skeletal muscle. Figure S3: Sphingolipid species along the de novo, sphingomyelinase (SMase), and salvage pathways in serum. Figure S4: Sphingolipid species of the cerebrosidase pathway in serum. Figure S5: Western blot full-length images of serum protein levels of APOA1 and PLIN4. Table S1: FSHD patients’ clinical data. Table S2: RT-qPCR probes utilized in the study. Table S3: MRM transitions. Table S4: Differentially expressed genes between experimental groups, reported as normalized RNA-seq read counts. Table S5: List of IPA canonical pathways significantly enriched in RNA-seq data.

## Abbreviations

The following abbreviations are used in this manuscript:

ANOVA	Analysis of variance
Cer	Ceramide
CTR	Control
CV	Coefficient of variation
dhCer	Dihydroceramide
dhSM	Dihydrosphingomyelin
ECM	Extracellular matrix
ER	Endoplasmic reticulum
ERAD	Endoplasmic reticulum-associated degradation
ESI	Electrospray ionization
EVs	Extracellular vesicles
FA	Fatty acid
FDR	False discovery rate
FSHD	Facioscapulohumeral dystrophy
GM1	Monosialotetrahexosylganglioside
GM3	Monosialodihexosylganglioside
HDL	High-density lipoprotein
HexCer	Hexosylceramide
HPLC	High-performance liquid chromatography
IPA	Ingenuity Pathway Analysis
IPP	Isopentenyl diphosphate
LacCer	Lactosylceramide
LC-ESI/MS	Liquid chromatography–electrospray ionization mass spectrometry
LC-MS	Liquid chromatography–mass spectrometry
LC-MS/MS	Liquid chromatography-tandem mass spectrometry
MRI	Magnetic resonance imaging
MRM-MS	Multiple reaction monitoring mass spectrometry
mTOR	Mechanistic target of rapamycin
PPP	Pentose phosphate pathway
RNA-seq	RNA sequencing
ROS	Reactive oxygen species
RT-qPCR	Reverse transcription quantitative PCR
S1P	Sphingosine-1-phosphate
SD	Standard deviation
SM	Sphingomyelin
SMase	Sphingomyelinase
Sph	Sphingosine
TCA cycle	Tricarboxylic acid cycle
TEM	Transmission electron microscopy
UPLC	Ultra-performance liquid chromatography
UPLC-MS/MS	Ultra-performance liquid chromatography–tandem mass spectrometry
UPR	Unfolded protein response
